# A Spatiotemporal Consistency-Enhanced Tightly Coupled LiDAR-Visual-Inertial SLAM Method for Complex Degraded Environments

**DOI:** 10.3390/s26196301

**Published:** 2026-10-05

**Authors:** Chenxi Li, Chuanwei Zhang, Jiazhong Li, Panjie Lei, Sidong Yang, Jiahao Li

**Affiliations:** 1 School of Mechanical Engineering, Xi’an University of Science and Technology, Xi’an 710054, China; 2 Institute of Traffic Information, Shaanxi College of Communications Technology, Xi’an 710018, China

**Keywords:** simultaneous localization and mapping, LiDAR-visual-inertial fusion, time-offset estimation, photometric consistency, degradation awareness, virtual constraints, loop-closure detection

## Abstract

Tightly coupled LiDAR-visual-inertial simultaneous localization and mapping (simultaneous localization and mapping, SLAM) systems are prone to observation distortion, weak state observability, and cumulative drift under weak-texture conditions, dynamic illumination, geometric degradation, multisensor time asynchrony, and long-term operation. To address these issues, we propose a spatiotemporal consistency enhancement method. The method is built upon the sequentially updated Error-State Iterated Kalman Filter (ESIKF) framework of FAST-LIVO2. First, using the Inertial Measurement Unit (IMU) clock as the unified reference, the LiDAR–IMU and Camera–IMU time offsets are jointly estimated online, and the estimates are fed back to point-cloud motion compensation and image reprojection. Second, through joint parameterization of vignetting, exposure time, and the camera response function, pixel intensity is inversely mapped to scene irradiance, while scale normalization and physical feasibility constraints are introduced to improve the identifiability of photometric parameters. Third, a LiDAR health indicator is established from the spectrum of a scale-normalized Hessian matrix, and direction-selective kinematic virtual observations are introduced under degeneration when motion is in a steady state and the innovation passes the gating threshold. Finally, low-frequency loop-closure constraints are generated using a ring-sector descriptor, rotation-invariant matching, and point-cloud geometric verification, while small-residual gating and progressive correction are adopted to reduce violation of the small-error assumption of ESIKF. Experimental results show that the proposed method achieves an overall RMSE of 0.379 m on the self-built visual and geometric degradation datasets; on the building-corridor loop-closure sequence, the average ATE decreases from 0.188 m with FAST-LIVO2 to 0.080 m; and in the unified ablation experiment, the average RMSE of the full system decreases from 0.153 m with FAST-LIVO2 to 0.117 m, a reduction of 23.5%. These results indicate that the enhancement modules are complementary in improving multisource observation consistency, degradation robustness, and long-term drift suppression.

## 1. Introduction

Simultaneous localization and mapping (SLAM) is one of the core enabling technologies for mobile robots, unmanned aerial vehicles, autonomous driving, and intelligent inspection systems to achieve environmental perception, autonomous navigation, and spatial understanding [1,2]. As robotic applications expand from structured indoor environments to real-world environments characterized by large-scale, long-duration operation, severe illumination changes, and structural degradation, SLAM systems must not only maintain high local state-estimation accuracy but also provide robustness and global consistency under complex degraded conditions. Early single-sensor SLAM methods have made significant progress in specific scenarios, but their performance is usually constrained by sensor physics and environmental conditions. Visual SLAM can exploit rich texture information for localization and mapping, but is sensitive to illumination changes, weak textures, motion blur, and occlusion [3,4,5,6,7]; LiDAR can provide high-precision 3D geometric structure and is insensitive to illumination, and is therefore widely used for high-precision localization [8,9,10,11,12], but in geometrically degraded scenarios such as long corridors, large planar surfaces, glass facades, and repetitive structures, point-cloud constraints may be insufficient to fully observe all system degrees of freedom. The IMU provides high-frequency motion priors and improves short-term motion prediction, but standalone integration rapidly drifts due to accumulated bias and noise. To overcome the limitations of single-sensor solutions, LiDAR-visual-inertial fusion SLAM has gradually become an important direction for multisensor state estimation. Early methods mainly adopted loosely coupled strategies, in which each sensor subsystem independently estimated its state and the resulting high-level poses were then fused in the backend [13,14]. Such methods are structurally clear and easy to implement, but because the fusion mainly operates on estimated outputs from individual subsystems rather than raw observations, they can suffer from information loss, insufficient treatment of error correlations, and inconsistent estimation when a subsystem fails. In contrast, tightly coupled LiDAR-visual-inertial SLAM jointly exploits LiDAR geometric constraints, camera texture and photometric constraints, and the continuous-time motion prior from the IMU within a unified optimization or filtering framework, thereby achieving more effective information complementarity at the observation level and becoming a mainstream direction for robust SLAM in complex environments [15,16,17,18,19]. Among them, the FAST-LIVO/FAST-LIVO2 family [20,21], as representative tightly coupled LiDAR-visual-inertial odometry frameworks, directly fuses point-cloud residuals and visual photometric residuals in a sequentially updated ESIKF and constructs a unified local LiDAR-visual map. It offers strong advantages in real-time performance, local estimation accuracy, and computational resource consumption, making it particularly suitable for UAV and embedded mobile-platform deployment. However, existing tightly coupled frameworks still face three key issues in real complex environments. First, multisensor systems develop time offsets due to different hardware clocks, driver pipelines, and buffering mechanisms, causing point-cloud undistortion, visual reprojection, and filter residual construction to be performed at incorrect physical times, thereby introducing systematic residual bias and reducing state-estimation consistency [22]. Second, existing direct visual constraints typically assume inter-frame photometric consistency, whereas real imaging is simultaneously affected by lens vignetting, exposure-time variation, and nonlinear camera response; raw pixel intensities therefore cannot reliably represent scene irradiance, weakening the ability of visual observations to compensate for LiDAR-degraded directions [23]. Third, existing systems mainly rely on visual constraints to maintain state estimation during LiDAR geometric degradation, but when visual observations are also degraded by insufficient illumination, sparse texture, or motion blur, the system may still drift or even diverge along weakly observable directions. At the same time, the ESIKF frontend mainly guarantees local continuity, while errors caused by IMU bias, local degradation, and repeated environments continue to accumulate during long-term operation, ultimately compromising global map consistency. To address these issues, this paper takes FAST-LIVO2 as the direct baseline and proposes a spatiotemporal consistency-enhanced tightly coupled LiDAR-visual-inertial SLAM method for complex degraded scenarios, jointly strengthening multisource temporal consistency, visual photometric consistency, robustness to geometric degradation, and long-term global consistency within a unified ESIKF framework. The main contributions are as follows:

(1) Building on existing online spatiotemporal calibration ideas, we design a unified state augmentation, observability gating, and feedback remapping mechanism for LiDAR–IMU and Camera–IMU time offsets within the sequentially updated ESIKF of FAST-LIVO2, so that time-offset estimation directly participates in point-cloud undistortion and visual reprojection.
(2) Building on existing camera-response/vignetting modeling, we jointly embed exposure, vignetting, and the camera response function into the direct photometric residual of FAST-LIVO2, and introduce scale normalization, a positivity constraint on vignetting, and a monotonicity constraint on the inverse CRF to improve the physical identifiability of photometric parameters under complex illumination.
(3) Building on existing Hessian-spectrum-based degradation criteria, we construct a LiDAR health indicator with rotation-translation scale normalization and whitening, and implement direction-selective kinematic virtual observations through steady-state confidence, innovation gating, and covariance adaptation, thereby avoiding the misclassification of legitimate lateral/vertical multirotor motion as zero velocity.
(4) Loop-closure candidate retrieval adopts the ring-sector descriptor concept of Scan Context. The modifications in this work are not focused on the descriptor itself, but on using it as a low-frequency candidate generator combined with point-cloud geometric verification, adaptive loop-closure covariance, small-residual gating, and progressive ESIKF correction, so as to balance long-term drift suppression and frontend continuity.

The remainder of this paper is organized as follows: Section 2 introduces multisource time-offset state augmentation, sign conventions, and observability gating; Section 3 presents the joint photometric model with normalization and physical constraints; Section 4 introduces the scale-normalized LiDAR health indicator and direction-selective virtual observations; Section 5 describes loop-closure candidate generation, geometric verification, and progressive correction; Section 6 presents independent validation, unified ablation, sensitivity, and computational-efficiency experiments; and Section 7 summarizes the paper and discusses the scope of applicability and limitations.

## 2. Online Estimation of Multisource Time Offsets and Observation Correction

In a LiDAR–Camera–IMU fusion localization and mapping system, the three sensor types typically generate timestamps through different hardware clocks and driver pipelines. If a fixed time offset or slowly varying synchronization error exists, the continuous-time motion prior provided by the IMU is inconsistent with the physical times corresponding to LiDAR and visual observations: point-cloud undistortion invokes pose segments from incorrect time intervals, and visual reprojection is also computed using incorrect poses. This ultimately appears as systematic residual bias during filter updates, leading to unstable convergence, increased drift, and local map blurring [24]. Therefore, the time offsets of LiDAR and the camera relative to the IMU are incorporated into the filter state as estimated parameters, while point-cloud sampling times, image exposure times, and the IMU propagation state participate in residual construction under the same time reference, with offline alignment implemented at the driver level [25], thereby establishing a correction mechanism that ensures consistency between the observation time and the state interpretation time.

### 2.1. Time-Offset Model and Augmented Filter State

Using the IMU clock as the unified reference clock, for the sensors $s\in \{L,C\}t_{s}\delta_{s}$ (denoted as LiDAR and Camera, respectively), the original timestamp be $t_{s}$ and the time offset relative to the IMU be $\delta_{s}$. The physical time corresponding to the observation in the IMU reference clock can then be written as:

(1) $$t=t_{s}-\delta_{s}$$

To explicitly characterize the influence of the time offset on the observation interpretation time, $\delta_{L}\delta_{C}$ are jointly augmented into the system state vector. Since synchronization errors can usually be approximated as constant over short time scales but may slowly drift during long-term operation due to driver latency, buffer jitter, or changes in the computational pipeline, a random-walk model is adopted as a prior constraint on the time offsets:

(2) $$\delta_{s,k+1}=\delta_{s,k}+w_{s,k},\;w_{s,k}∼N\left(0,\sigma_{\delta_{s}}^{2}\right)$$

where $\sigma_{\delta_{s}}^{2}$ denotes the random-walk variance and controls the prior strength of the time-offset variation. On the one hand, this allows online tracking of slowly varying synchronization offsets; on the other hand, it prevents other factors such as extrinsic-calibration errors or bias errors from being incorrectly absorbed into the time-offset parameters. $\sigma_{\delta_{s}}^{2}$.

### 2.2. Filter Update and Observation Reconstruction Correction

For a sensor observation at an arbitrary time $z_{s}$, its measurement equation can be uniformly written in the form of “evaluating the state at the shifted time”:

(3) $$z_{s}(t_{s})=h_{s}(x(t_{s}-\delta_{s}))+v_{s}$$

where $h_{s}\left(\cdot \right)$ denotes the observation function of sensor s, x(⋅) denotes the system state at the corresponding time, and $v_{s}$ denotes the measurement noise.

Thus, the time offset is not an independently added perturbation to the measurement; instead, it enters the measurement model by changing the state interpretation time associated with the observation. Consequently, the observation residual is related not only to the state error but also directly coupled with the time-offset error. When the time offset $\delta_{s}x(t_{s}-\delta_{s})$ is on the millisecond scale, a first-order approximation can be made at the current linearization point:

(4) $$x(t_{s}-\delta_{s})\approx x(t_{s})-ẋ(t_{s})\delta_{s}$$

Using the first-order time derivative of the state, the state change induced by the time shift is converted into an explicitly estimable linear correction. Further linearization of the observation function gives:

(5) $$h_{s}(x(t_{s}-\delta_{s}))\approx h_{s}(x(t_{s}))-H_{x}ẋ(t_{s})\delta_{s}$$

where H_x_ is the Jacobian of the observation function with respect to the system state, and $\dot{x}$(t_s_) is the first-order time derivative of the state. We define the corrected physical time as t* = t_s_ − δ_s_; therefore, the time-offset Jacobian must have a negative sign. The actual ESIKF implementation is consistent with Equation (5) and uses −H_x_$\dot{x}$(t_s_).

(6) $$H_{\delta }=\partial h_{s}∕\delta_{s}\approx -H_{x}ẋ(t_{s})=-(d/dt)h_{s}(x(t))│t_{s}$$

Accordingly, when constructing the extended Kalman filter update, the online estimates of ${\hat{\delta }}_{L}{\hat{\delta }}_{C}$ can be obtained simultaneously with the correction of the pose and biases.

To avoid strong correlation between the time offsets and the pose/IMU biases under low-excitation motion, a local observability check is further performed on the time-offset sub-block. Let the stacked whitened Jacobian over a sliding window be Jw, and extract the time-offset columns and the associated inertial-state columns to form Jt. When the minimum singular value of Jt falls below a threshold or its condition number exceeds a threshold, updates of δL and δC are suspended and only random-walk propagation is maintained. Only when the window contains sufficient angular-velocity variation, linear-acceleration variation, and nondegenerate geometric/image gradients can the time-offset columns remain sufficiently linearly independent of the pose, velocity, and bias columns. During constant-velocity straight motion, nearly constant angular velocity, or simultaneous texture/geometric deficiency, the time offset is locally unobservable; the above gating is therefore used to prevent other state errors from being incorrectly absorbed.

In the convergence test with intentionally perturbed initial values, the LiDAR–IMU and Camera–IMU initial offsets were set to ±20 ms and ±30 ms around the ground truth, respectively. The results show that on sequences with normal six-degree-of-freedom excitation, both types of time offset converge to within ±1.5 ms in approximately 8–15 s. During low-excitation segments, the estimates remain frozen and continue converging after sufficient motion excitation is restored.

After the filtering process is completed, the estimated time offsets are fed back into the original timestamp remapping process:

(7) $${\hat{t}}_{s}=t_{s}-{\hat{\delta }}_{s}$$

**Figure 1:**
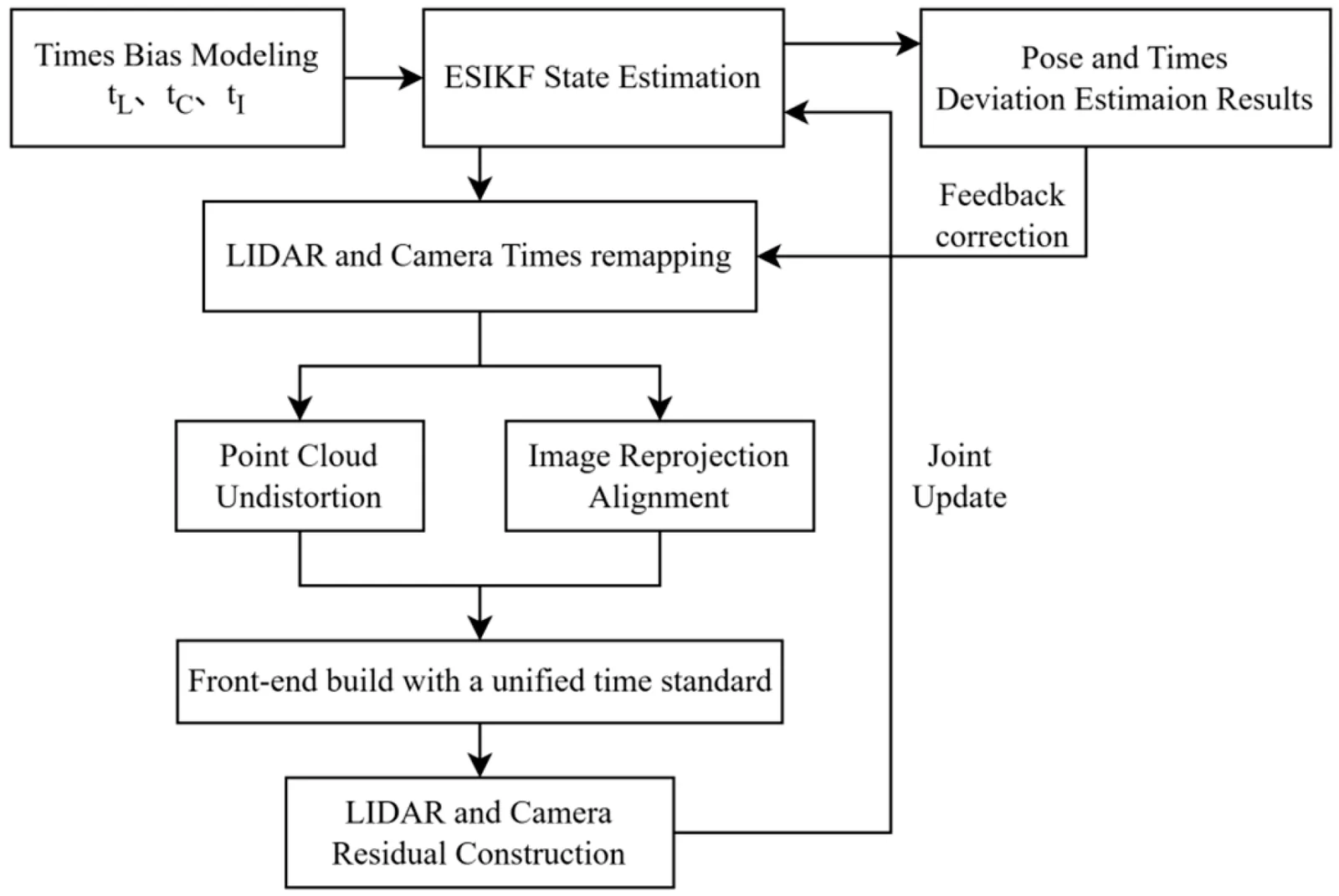
Time-offset feedback correction pipeline.

The LiDAR point cloud is motion-compensated using the corrected sampling time, while image observations are modeled through reprojection using the corrected exposure time. Meanwhile, continuous-time IMU propagation provides the rotation and translation poses at the same reference time. This realizes a feedback correction pipeline of “online time-offset estimation–timestamp remapping–observation reconstruction–filter update,” as shown in Figure 1. Compared with methods relying only on hardware synchronization or offline timestamp correction, this pipeline estimates the time offsets online as state variables and directly applies them to the construction of tightly coupled frontend observations.

## 3. Visual Photometric Consistency Enhancement

A key component of direct visual-inertial odometry is photometric error minimization: state estimation is achieved by optimizing the difference between the observed pixel intensities and the pixel values predicted from camera motion and scene geometry [26]. However, in real imaging, pixel intensity is not a linear representation of scene irradiance. On the one hand, lens vignetting and nonlinear camera response cause energy attenuation or intensity compression toward the image periphery [27]; on the other hand, adaptive exposure-time changes with illumination alter the scale of pixel intensity, invalidating the assumption of inter-frame photometric consistency. Directly using uncorrected pixel intensities to construct photometric residuals introduces systematic bias and further contaminates ESIKF state updates. Therefore, this paper performs online photometric calibration to inversely map pixel intensities to the actual scene irradiance, thereby restoring inter-frame consistency.

### 3.1. Joint Photometric Model Construction

**Figure 2:**
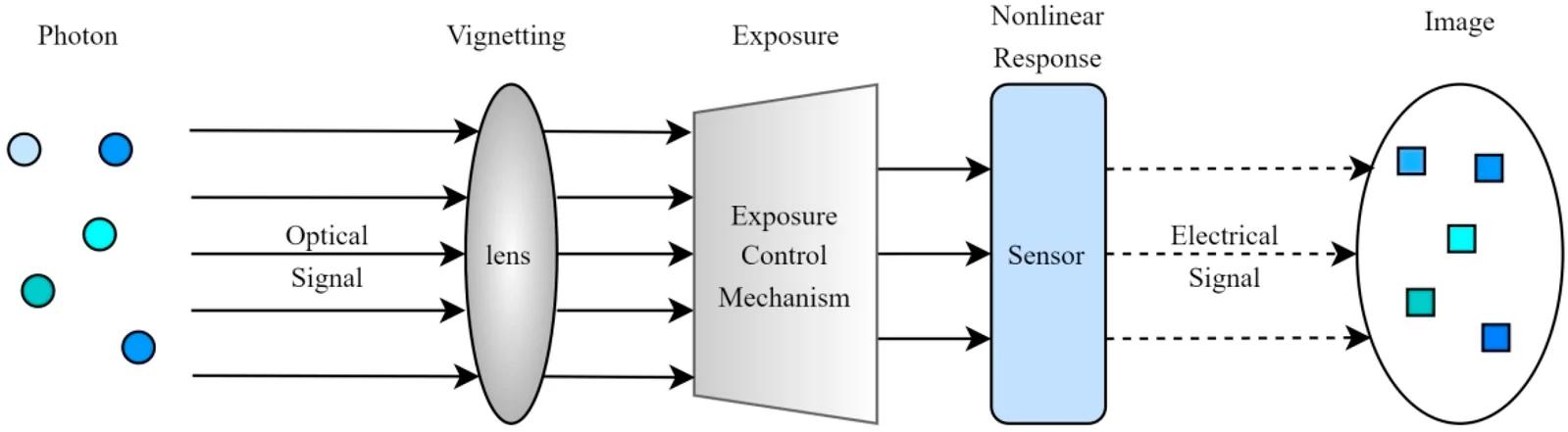
Imaging process.

The camera imaging process can be regarded as a mapping in which optical signals undergo multiple physical transformations before being converted into digital signals, as shown in Figure 2.

For the observed intensity uI(u) at the pixel coordinate in the image plane, its physical model can be expressed as:

(8) I(u)=f(t⋅V(u)⋅B(u))

where I(u) denotes the observed intensity at pixel coordinate u, B(u) denotes the scene irradiance, V(u) is the lens vignetting function that characterizes energy attenuation with increasing radial distance as light passes through the lens, t is the exposure time that determines the total amount of optical energy accumulated at the sensor, and f(⋅) is the camera response function (CRF) that nonlinearly maps the accumulated optical energy to digital intensity values.

To use this model in optimization, we need its inverse form, namely, to recover scene-structure-related irradiance from the observed pixel value IB(u):

(9) $$B\left(u\right)=\frac{f^{-1}\left(I\left(u\right)\right)}{t\cdot V\left(u\right)}$$

To embed this inverse mapping into ESIKF while maintaining solvability, the vignetting function $V\left(u\right)tf^{-1}\left(\cdot \right)$, exposure time, and response function need to be parameterized in a low-dimensional form:

(1) Higher-Order Polynomial Representation of the Vignetting Function

Given that most lens vignetting is approximately radially symmetric [28] and that attenuation is mainly dominated by even-order radial terms, we describe the logarithmic vignetting using an even-order polynomial with a maximum degree of 4, i.e., including r^2^ and r^4^ terms. An exponential mapping is then used to ensure that the vignetting function remains positive:

$$V(r)\;=\;\exp(a_{2}r^{2}\;+\;a_{4}r^{4}),V\left(r\right)=\exp\left(a_{2}r^{2}+a_{4}r^{4}\right)$$

(10) V(0)=1

where r denotes the normalized radial distance from the pixel to the principal point, and $a_{2}$ and $a_{4}$ are the vignetting parameters to be estimated, which are incorporated into the ESIKF state vector for online estimation.

(2) Inverse Parameterization of Exposure Time

To improve the linearity of the photometric residual equation and ensure numerical stability, the inverse of the exposure time $\tau =t^{-1}k\;and\;k+1$ is included in the state vector and estimated within ESIKF. Because exposure time at adjacent instants usually exhibits continuity, a random-walk process is therefore used to describe its evolution:

(11) $$\tau_{k+1}=\tau_{k}+n_{\tau },\;n_{\tau }∼N\left(0,\sigma_{\tau }^{2}\right)$$

where ${\;n}_{\tau }$ is the process noise of the exposure adjustment.

(3) Grossberg-Nayar Empirical Model Parameterization of the Camera Response Function (CRF)

The inverse function of the CRF, g(I) = f^−1^(I), can be regarded as a photometric correction function. A simple Gamma model has difficulty balancing representational capacity and generalization under complex illumination. Grossberg and Nayar [29] investigated the space of camera response functions using a database of real camera responses and provided a low-dimensional representation based on principal-component basis functions. Inspired by their PCA-basis representation, we construct the photometric correction function as a linear combination of the mean response and a small number of feature basis functions:

(12) $$g\left(I\right)=g_{0}\left(I\right)+\sum_{k=1}^{N}c_{k}h_{k}\left(I\right)$$

where g_0_(I) is the mean response function, h_k_(I) is the k-th feature response basis function, and c_k_ is the coefficient to be estimated. We use N = 4 response basis functions, converting the estimation of a complex nonlinear function into the estimation of a small number of coefficients. To eliminate the scale gauge freedom among exposure, vignetting, and the CRF, the inverse-exposure scale of the reference frame is fixed to 1, and g(0) = 0 and g(1) = 1 are enforced, thereby ensuring positive vignetting and a monotonically increasing inverse CRF.

The above normalization means that the system estimates only relative exposure and relative irradiance scales and does not claim to recover absolute radiometric quantities. If a lux meter is available for external calibration, a single scale factor is used only during experimental evaluation to map relative irradiance to the illuminance reference; this scale factor is not fed back into the filter state.

### 3.2. Irradiance Residual and Jacobian Derivation

For the same 3D map point p observed in the reference frame i and the current frame j, let the corresponding projected pixels be $u_{i}$ and $u_{j}$, and their observed gray levels be $I_{i}\left(u_{i}\right)$ and $I_{j}\left(u_{j}\right)$, respectively. The irradiance residual is defined as the difference between the irradiance of the point in the reference frame and that in the current frame:

(13) $$r_{p}=B_{i}-B_{j}=\tau_{i}\frac{g\left(I_{i}\left(u_{i}\right)\right)}{V\left(u_{i}\right)}-\tau_{j}\frac{g\left(I_{j}\left(u_{j}\right)\right)}{V\left(u_{j}\right)}$$

**Figure 3:**
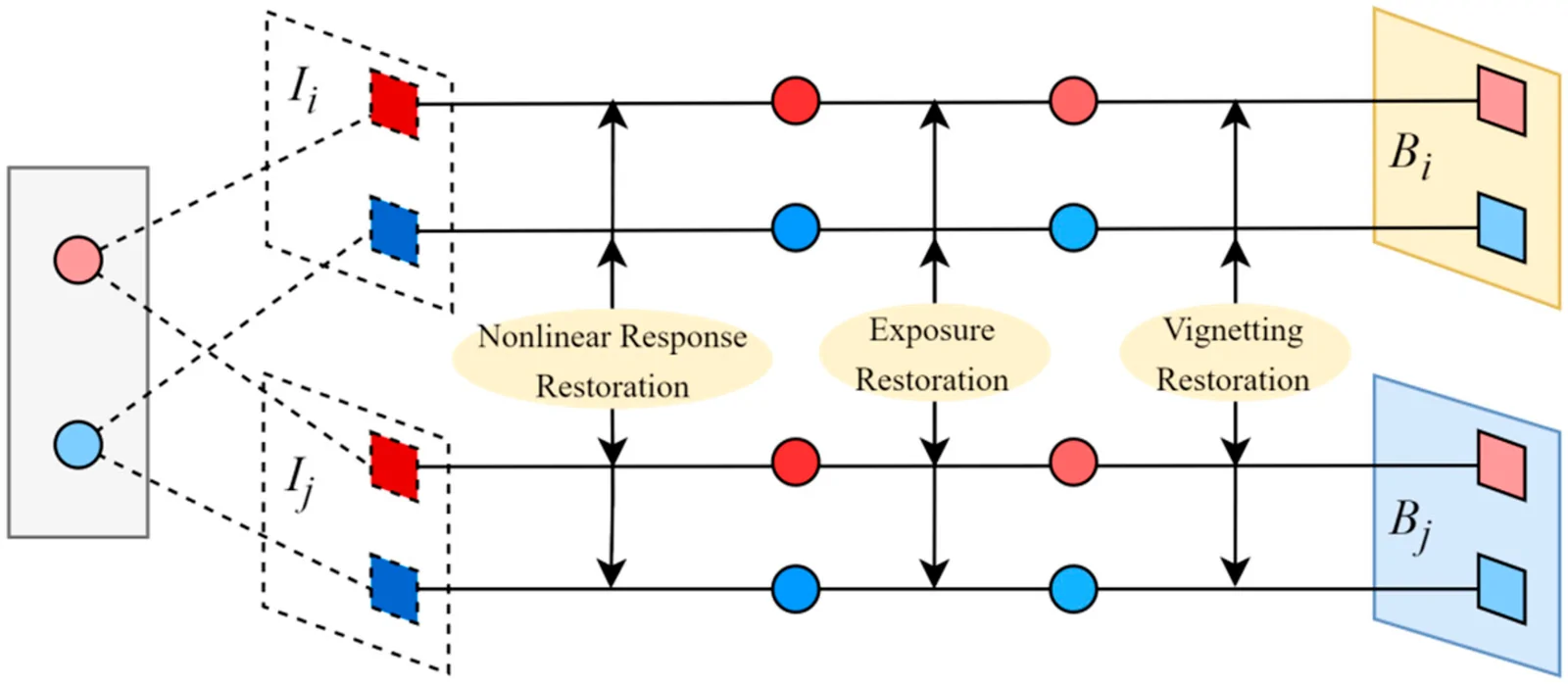
Principle of irradiance-residual formation and computation. The upper and lower branches represent the observations in the reference frame i and the current frame j, respectively. $I_{i}$ and $I_{j}$ denote the corresponding observed image intensities, while $B_{i}$ and $B_{j}$ denote the recovered scene irradiances. The arrows indicate the inverse photometric correction process, including nonlinear response restoration, exposure restoration, and vignetting restoration. The red and blue markers illustrate different observations used to demonstrate the irradiance restoration process. Dashed lines indicate the projection correspondences between the 3D map points and their image observations.

In this form, the photometric residual depends only on the true scene irradiance, thereby ensuring global photometric consistency. The formation principle of the irradiance residual is illustrated in Figure 3.

To minimize this residual in the Kalman filter, its Jacobian with respect to the augmented state vector $J_{p}\delta x\delta x_{p}$, including state errors and photometric-parameter errors, must be derived. According to the chain rule, the overall Jacobian can be written as:

(14) $$J_{p}=\frac{\partial r_{p}}{\partial \delta x}=\frac{\partial r_{p}}{\partial u}\cdot \frac{\partial u}{\partial \delta x}+\frac{\partial r_{p}}{\partial \delta x_{p}}$$

where the geometric term $\frac{\partial r_{p}}{\partial u}\nabla I\nabla V$ involves the image gradient and the vignetting-function gradient and can be expressed as:

(15) $$\frac{\partial }{\partial u}\left(\tau \frac{g\left(I\left(u\right)\right)}{V(u)}\right)=\tau \frac{V(u)\cdot g\prime \left(I\right)\nabla I(u)-g\left(I\right)\nabla V(u)}{{V(u)}^{2}}$$

By explicitly introducing a correction term in the geometric component to subtract the vignetting gradient, the optimizer can be prevented from misinterpreting illumination attenuation caused by vignetting as a change in scene texture, thereby improving the physical consistency of the estimate ∇V.

## 4. Geometric Degradation Awareness and Adaptive Fusion of Virtual Constraints

### 4.1. Lidar Health Quantification Based on the Hessian Spectrum

In SLAM systems, “degradation” essentially means that sensor observations are insufficient to constrain all degrees of freedom of the system state, causing divergence along unconstrained directions. To enable measurable online detection, we build a degradation quantification framework based on Hessian-matrix eigenvalues, drawing on the spectral analysis of degradation mechanisms in optimization-based state estimation by Zhang et al. [30] and the online degradation criterion proposed by Zhou et al. [31], and use a unified mathematical scale to evaluate LiDAR health.

In nonlinear least squares, the Hessian matrix H can be approximated by the Fisher information matrix to characterize the strength of observation constraints on the state variables:

(16) $$H=J^{⊤}\Sigma^{-1}J$$

where J is the Jacobian of the observation residual with respect to the state, and Σ is the measurement-noise covariance. For the i-th point, the Jacobian of the point-to-plane residual can be expressed as:

(17) $$J_{i}=n_{i}^{⊤}\frac{\partial Tp_{i}}{\partial \delta x}$$

where $n_{i}$ is the corresponding plane normal vector, $p_{i}$ is the 3D point coordinate, and δx is the pose increment. Therefore, the approximate Hessian matrix for the point-to-plane residual can be written as:

(18) $$H_{L}=\sum_{i=1}^{N}J_{L,i}^{T}\Sigma_{L}^{-1}J_{L,i}$$

The matrix is then eigendecomposed, and the eigenvalues are arranged in descending order:

(19) $$H_{L}=V\Lambda V^{⊤},\;\Lambda =diag\left(\lambda_{1},\lambda_{2},\lambda_{3},\lambda_{4},\lambda_{5},\lambda_{6}\right)$$

Because $H_{L}$ is a symmetric positive semidefinite matrix, its eigenvalues and eigenvectors correspond to observability in different directions of the state space: larger eigenvalues indicate stronger constraints in the corresponding directions, with point-to-plane residuals varying more strongly with state changes; smaller eigenvalues indicate weaker constraints and significant ambiguity. Considering that eigenvalues are affected by point-cloud quantity and environmental scale, we combine absolute and relative constraint strengths to construct a normalized LiDAR health indicator.

Because rotational increments (rad) and translational increments (m) have different physical units, we do not directly compute the spectrum of the raw 6-DoF Hessian. First, we construct the scale matrix S = diag(Lc, Lc, Lc, 1, 1, 1) using the characteristic scale Lc of the current local point cloud (the median distance from valid points to the sensor), and whiten the residuals according to the measurement covariance, obtaining $\bar{J}$ = Σ^−1^/^2^JS^−1^ and $\bar{H}$ = $\bar{J}$ᵀ$\bar{J}$. The subsequent absolute strength and condition ratios are both computed from the eigenvalues of $\bar{H}$, thereby reducing the direct influence of coordinate scale, point count, and residual-weight changes on the threshold.

(20) $$\alpha_{L}=𝒮\left(\lambda_{min};\theta_{a}\right)\cdot 𝒮\left(\frac{\lambda_{min}}{\lambda_{max}};\theta_{r}\right)$$

where ηabs is the whitened and scale-normalized absolute constraint strength, ηrel is the relative constraint strength corresponding to the minimum/maximum eigenvalue ratio; τα and τρ are the absolute and relative thresholds, respectively; and kσ is the Sigmoid slope. The health indicator is used only for continuous weighting and trigger decisions and is not treated as a physical quantity directly comparable across sensors.

(21) $$𝒮\left(x;\tau \right)=\frac{1}{1+exp\left(-k\left(x-\theta \right)\right)}$$

### 4.2. Motion-State-Gated Direction-Selective Virtual Observation Update

**Figure 4:**
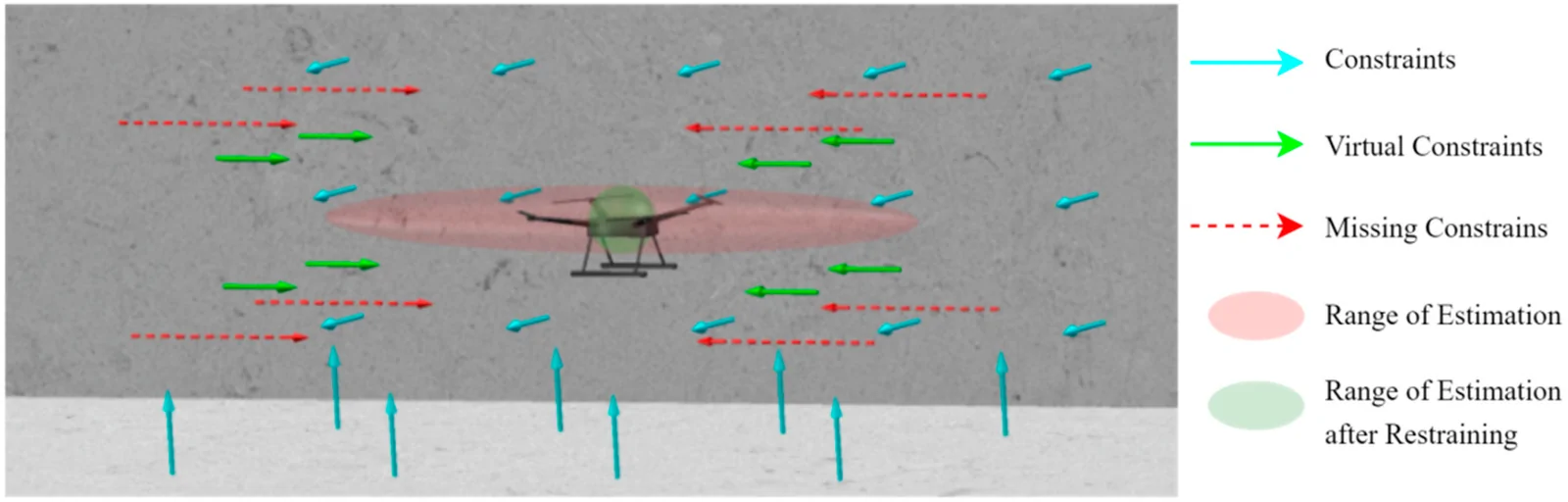
Schematic of the virtual-constraint principle. The UAV shown in the center represents the platform to which the direction-selective virtual constraints are applied according to its motion state.

In extreme environments, both LiDAR and visual observations may simultaneously lack effective constraints, and relying only on IMU integration causes localization error to accumulate rapidly. It should be emphasized that multirotor UAVs do not satisfy the strict nonholonomic constraint (NHC) in the sense used for ground vehicles: lateral and vertical velocities may both be nonzero during turning, wind compensation, and attitude changes. Therefore, in this work, NHC is treated only as a source of “candidate kinematic pseudo-observations” and is instead implemented as a direction-selective, state-gated weak constraint, rather than forcibly setting body-frame lateral/vertical velocities to zero. The principle of the proposed direction-selective virtual constraint is illustrated in Figure 4.

A sliding-window steady-state detector and innovation consistency are jointly used to determine whether a virtual observation is enabled. Only when the specific-force variance, mean angular velocity, and velocity change rate within the window are all below their thresholds, and the predicted motion in the candidate direction is close to steady state, is that direction allowed to enter the selection matrix. Once obvious lateral acceleration, vertical maneuvering, turning, or wind disturbance is detected, the corresponding direction is immediately removed from the selection matrix.

Let the sliding-window length be $NE_{a}E_{\omega }$. If the variance of the acceleration measurements within the window is below a threshold, this indicates that the body does not undergo significant velocity changes; if the mean angular-velocity magnitude within the window is below a threshold, this indicates that the body is not undergoing substantial attitude adjustment. The steady-state decision provides the physical basis for introducing NHC and constant-velocity (CV) virtual observation residuals, allowing them to be incorporated into the ESIKF update [32].

Let the instantaneous linear velocity be expressed in the IMU frame and in the world frame, respectively. The two representations satisfy the following transformation relationship:

(22) $$v^{I}={\left(R_{I}^{G}\right)}^{⊤}v^{G}$$

where the rotation matrix maps vectors from the IMU frame to the world frame.

Under the steady-state assumption, a time-varying selection matrix Sᵥ(t) is constructed to extract only the velocity components that pass the steady-state test, and the weak virtual-observation residual is defined as:

(23) $$r_{NHC}=D\cdot v^{I}=D\cdot {\left(R_{I}^{G}\right)}^{⊤}v^{G}-z_{NHC}$$

where $z_{NHC}$ is the desired virtual observation value (typically a zero vector). When the estimated velocity drifts along a stable direction, this residual provides feedback correction to the filter state.

Furthermore, after constraining the stable directions, the system velocity variation must also be constrained. Under the constant-velocity assumption, the true velocity at the current time $v_{k}^{\left(G\right)}v_{k-1}^{\left(G\right)}r_{CV}$ is expected to be close to the estimated velocity at the previous time. A constant-velocity observation is therefore constructed, with the observation residual given by:

(24) $$r_{CV}\;={\;v}_{k}^{\left(G\right)}-v_{k-1}^{\left(G\right)}$$

The Jacobians of the two virtual-observation models with respect to the error-state variables are derived separately: δx.

(25) $$H_{NHC}=\partial r_{NHC}/\partial \delta x,H_{CV}=\partial r_{CV}/\partial \delta x$$

To prevent false steady-state detection from making the filter overconfident, the covariance of the virtual observations is not set to a fixed small value but is jointly adjusted according to LiDAR health and steady-state confidence. Meanwhile, a χ^2^ threshold test is applied to the normalized innovation squared, and the pseudo-observation is rejected when the threshold is not passed. In the disturbance experiment, after lateral acceleration/wind disturbance was deliberately introduced during degraded segments, fixed NHC increased the ATE from 0.684 m to 0.731 m, whereas the proposed gating strategy yielded 0.492 m, with a pseudo-constraint false-trigger rate of approximately 2.8%.

When the system is under extremely low-observability conditions and satisfies the steady-state criterion, the above two terms are included as observation terms in the ESIKF update, thereby using kinematic priors to suppress the growth of localization error from rapid accumulation to approximately linear growth and improving system stability when constraints are missing ${\;\alpha }_{L}$.

## 5. Loop-Closure Detection and Global Consistency Optimization

**Figure 5:**
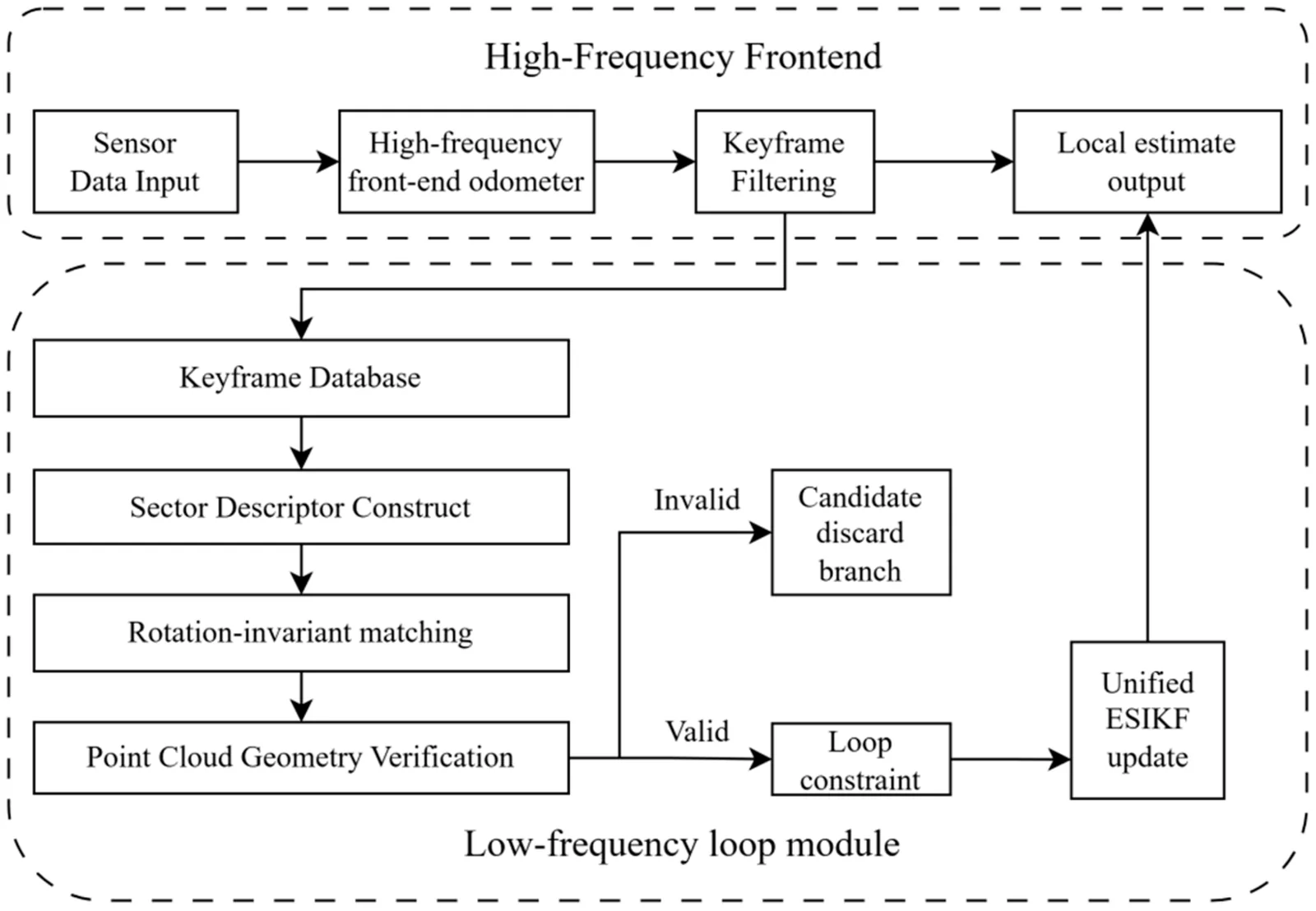
Loop-closure constraint generation and state-update process.

FAST-LIVO2 relies on a tightly coupled LiDAR-visual-inertial frontend to provide continuous and accurate pose estimation at the local scale. However, when the system relies only on recursive updates from local observations, residual IMU biases, local geometric degradation, and time-varying noise in visual and LiDAR observations can still cause errors to accumulate over time, resulting in pronounced global drift in large-scale or long-term mapping tasks [33]. Therefore, a low-frequency, independently running loop-closure detection module is introduced in addition to the FAST-LIVO2 frontend. The frontend odometry generates a sequence of keyframes in chronological order. When the current keyframe and a historical keyframe are determined through scene retrieval and geometric verification to be repeated observations of the same place, a loop-closure edge is formed between them across time. This loop-closure edge and the local pose edges generated by continuous frontend recursion jointly form a closed constraint loop for correcting the accumulated pose drift between the two keyframes. The loop-closure constraint generation and state-update process are shown in Figure 5.

### 5.1. Keyframe Extraction Strategy

Keyframes are used instead of ordinary frames as the objects for loop-closure retrieval and constraint construction, in order to control the size of the historical database and reduce the probability of false matches. Let $t_{k}$ and $\psi_{k}$ denote the translation vector and heading angle of the current frame, respectively, and let $t_{k_{c}}$ and $\psi_{k_{c}}$ denote those of the most recently inserted keyframe. If the following condition is satisfied, the current frame is selected as a new keyframe:

(26) $${∥t_{k}-t_{k_{c}}∥}_{2}>\tau_{t}\;or\;|\psi_{k}-\psi_{k_{c}}|\;>\tau_{\varphi }$$

where $t_{k}$ and $t_{k_{c}}$ are the translation vectors of the current frame and the most recently inserted keyframe, respectively; $\psi_{k}$ and $\psi_{k_{c}}$ are their corresponding heading angles; and $\tau_{t}$ and $\tau_{\varphi }$ denote the translation and heading-change thresholds, respectively. By jointly using translation and heading information, the system can retain sufficient scene-change characteristics while controlling the database size, thereby providing a sparse yet sufficiently comprehensive candidate set for subsequent descriptor retrieval $t_{k}t_{k_{c}}\psi_{k}\psi_{k_{c}}\tau_{t}\tau_{\varphi }$.

### 5.2. Ring-Sector Descriptor Construction

To rapidly screen historical candidate frames, a Scan Context-type ring-sector descriptor [34] is used to encode keyframe point clouds structurally. The ring-sector partitioning, maximum-height encoding, and cyclic sector shifts all follow existing Scan Context ideas; they are not claimed as new contributions in this work. Instead, the descriptor’s low-cost place-retrieval capability is used to provide candidates for subsequent geometric verification and filter-based global correction.

Given a point cloud frame

$$P={\left\{p_{n}\right\}}_{n=1}^{N}$$

where $p_{n}=\left(x_{n},y_{n},z_{n}\right)$ denotes the n-th point in the sensor coordinate system, each point is converted from Cartesian coordinates to polar coordinates as

(27) $$\begin{array}{cccc} & \begin{array}{c} \rho_{n}=\sqrt{x_{n}^{2}+y_{n}^{2}},\\ \theta_{n}=atan2\left(y_{n},x_{n}\right). \end{array} & & \end{array}$$

where $\rho_{n}$ denotes the horizontal distance from the n-th point to the sensor origin, and $\theta_{n}$ denotes its azimuth angle in the horizontal plane.

**Figure 6:**
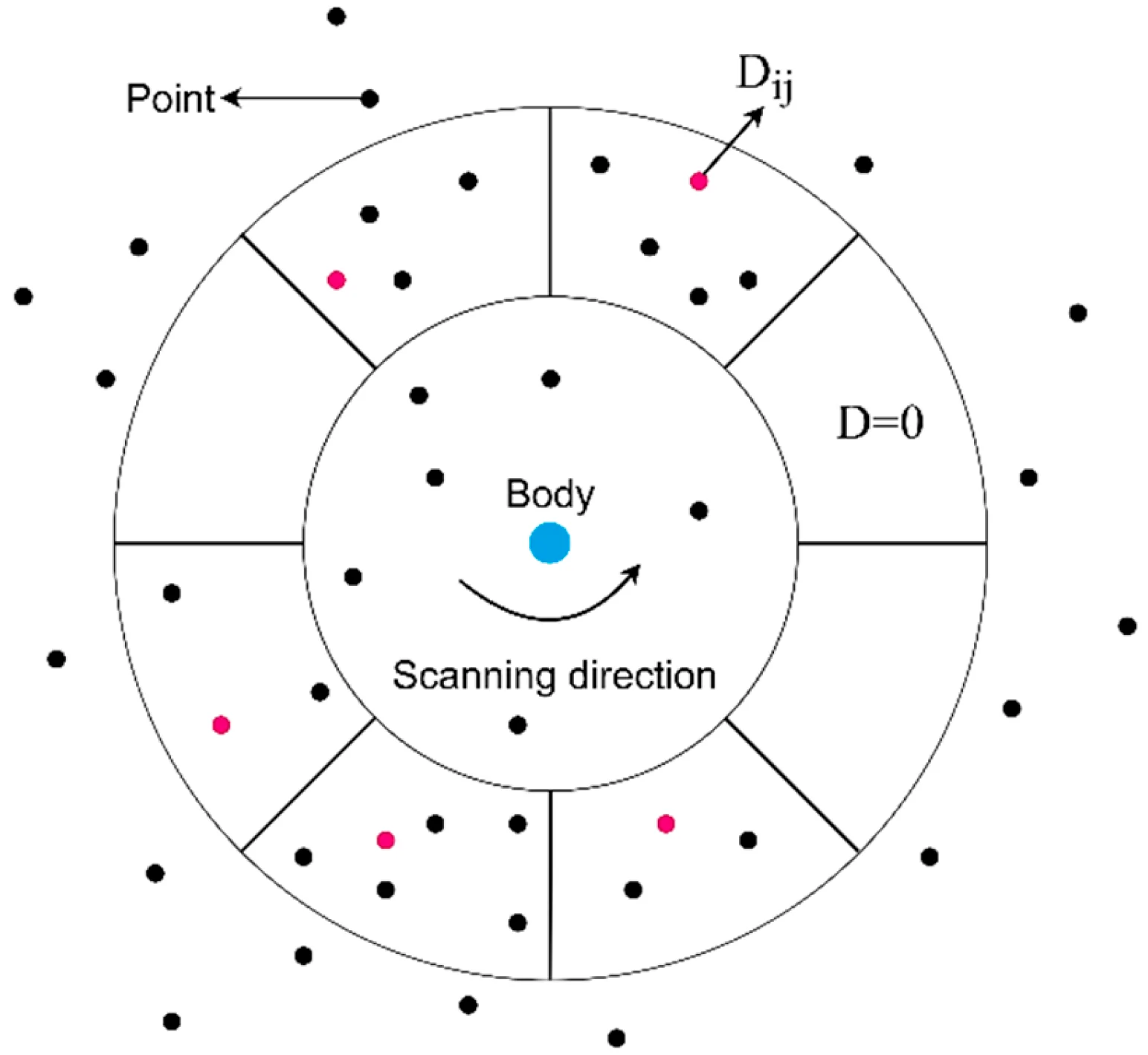
Sector descriptor and maximum-height feature.

The horizontal plane is then divided into $N_{r}$ concentric rings and $N_{s}$ sectors. For each point $p_{n}$, its corresponding ring and sector indices are denoted by i and j, respectively. The descriptor element $D_{ij}$ is defined as the maximum height among all points falling within the i-th ring and j-th sector:

(28) $$D_{ij}=\{\begin{array}{cc} \underset{p_{n}\in B_{ij}}{max}z_{n}, & B_{ij}\neq ⌀,\\ 0, & B_{ij}=⌀, \end{array}$$

where $B_{ij}$ denotes the set of points within the grid cell corresponding to the i-th ring and j-th sector. Using maximum height as the cell feature preserves vertical contour information with low descriptor dimensionality and improves the discriminability between scenes at different locations, as shown in Figure 6.

To reduce the influence of different measurement distances and point-cloud sparsity on similarity computation, the descriptor matrix is normalized ring by ring. This treatment gives near, dense regions and far, sparse regions more balanced weights during matching, thereby improving the stability of candidate loop-closure retrieval.

Because the maximum-height feature is sensitive to occlusion, dynamic objects, and local geometric changes, short-term unstable point clusters are removed before actual retrieval, and upper-quantile clipping is applied to cell heights. Candidates are used only for coarse retrieval; final acceptance must be jointly verified using point-cloud registration residuals, the number of valid correspondences, and the time interval. Perturbation tests show that Recall@1 is approximately 0.94/0.91/0.86 under random point losses of 10%/20%/30%, respectively, and approximately 0.89 when 10% dynamic-point interference is introduced.

Let $d_{i}$ denote the i-th row of the descriptor matrix D, and define its normalized row vector $d_{i}^{\prime }$ as

(29) $$d\prime_{i}=\frac{d_{i}}{{∥d_{i}∥}_{2}\;+\;\epsilon }$$

where $∥\cdot {∥}_{2}$ denotes the Euclidean norm and ϵ is a small positive constant introduced to avoid division by zero.

### 5.3. Rotation-Invariant Matching

Because the FAST-LIVO2 frontend uses the IMU gravity direction to align the point cloud with gravity, the main viewpoint difference when revisiting the same scene is concentrated in the heading-angle offset about the vertical axis. Based on this property, cyclic-shift search is performed only along the sector dimension, reducing candidate matching computation while maintaining rotation invariance.

Let the current and historical frames correspond to the current and historical descriptors, respectively. For each candidate cyclic shift, the historical descriptor is cyclically shifted to the right by the corresponding number of columns along the sector dimension, and their ring-wise distance is computed. The descriptor distance between the current and historical frames is defined as:

(30) $$\underset{k\in \{0,\ldots ,N_{s}-1\}}{min}\frac{1}{N_{r}}\sum_{i=1}^{N_{r}}{∥d_{i}^{\left(q\right)}-{𝒮}_{k}\left(d_{i}^{\left(h\right)}\right)∥}_{2}$$

where $d_{i}^{\left(q\right)}d_{i}^{\left(h\right)}ik^{*}$ and denote the -th row vectors of the current and historical descriptors, respectively. The shift with the minimum distance is then selected as the optimal shift for alignment:

(31) $$k^{*}=\underset{k\in \{0,\ldots ,N_{s}-1\}}{min}\frac{1}{N_{r}}\sum_{i=1}^{N_{r}}{∥d_{i}^{\left(q\right)}-{𝒮}_{k}\left(d_{i}^{\left(h\right)}\right)∥}_{2}$$

The relative heading estimate between the current and historical frames is then obtained as:

(32) $$\Delta \psi \approx \frac{2\pi k^{*}}{N_{s}}$$

With this mechanism, even when the UAV revisits the same region with a different heading, the system can obtain a stable similarity measure through cyclic sector matching and provide a reliable initial heading estimate for subsequent geometric registration.

### 5.4. Loop-Closure Decision, Constraint Construction, and Adaptive Weighting

After obtaining candidate matches at the descriptor level and an initial relative heading, the system performs point-cloud geometric registration between the current keyframe and candidate historical keyframes. The average geometric residual after registration, the number of valid correspondences, and the time interval between candidate frames are jointly used as the criteria for loop-closure acceptance, reducing false closures caused by repetitive structures.

Let the number of valid correspondences obtained after registration between the current frame and a historical keyframe be N, and let $r_{i}$ denote the geometric residual of the i-th correspondence. The average matching residual between the current frame and the keyframe is defined as:

(33) $$E=\frac{1}{N}\sum_{i=1}^{N}r_{i}^{2}$$

where $r_{i}$ takes the form of the point-cloud residual used in the tightly coupled frontend optimization. When this is below the threshold and the number of valid matches satisfies the requirement, the system determines that the current and historical keyframes form a valid loop closure. In this case, the loop closure does not directly replace the frontend pose. Instead, a loop-closure observation is constructed from the relative pose obtained by registering the point clouds of the two frames. Let the frontend-predicted pose at the current time be $T_{k}$, and let the corrected pose obtained from the loop-closure constraint be $T_{L}$. The loop-closure pose residual is then defined as:

(34) $$r_{L}=Log(T_{L}T_{k})$$

where Log(⋅):SE(3)→se(3) denotes the logarithmic map from the Lie group SE(3) to its Lie algebra se(3), and the residual vector contains both translational and rotational errors. To incorporate the loop-closure constraint into ESIKF, let x denote the current nominal system state and δx denote the corresponding error state. A first-order linearization of the nonlinear residual at the current estimate gives:

(35) $$r_{L}\left(x⊕\delta x\right)\approx r_{L}\left(x\right)+H_{L}\delta x$$

where $H_{L}$ is the Jacobian of the loop-closure residual with respect to the error state. After incorporating the constant term into the observation vector, the standard linear observation form is obtained:

(36) $$z_{L}\approx H_{L}\delta x_{k}+n_{L}$$

where $H_{L}$ is the Jacobian of the loop-closure observation with respect to the error state, and $n_{L}$ denotes the corresponding observation noise. Because different loop-closure matches have different reliability, a fixed weight is not used for all loop-closure constraints. Instead, the observation-noise covariance matrix is adaptively adjusted according to the average point-cloud matching residual between the current and candidate keyframes. The loop-closure observation-noise covariance is defined as:

(37) $$\Sigma_{L}(E)=\alpha EI_{6}+\Sigma_{min}$$

where α>0 is a proportionality coefficient, $I_{6}$ is the 6×6 identity matrix, and $\Sigma_{min}$ is a covariance lower bound introduced for numerical stability. This design makes the weight of the loop-closure constraint adaptively determined by the average point-cloud matching residual: the smaller the matching residual, the larger the weight of the loop-closure observation in the overall residual optimization, allowing the current state to converge more effectively toward the globally consistent solution.

When jointly updated with the frontend observations, the final objective solved by the system can be expressed as a weighted residual-minimization problem:

(38) $$\underset{\mathrm{\delta x}}{\min}\left(\sum_{s\in \{L,C\}}∥r_{s}{(\delta x)∥}_{P_{s}}^{2}+∥r_{L}{(\delta x)∥}_{\Sigma_{L}\left(E\right)}^{2}\right)$$

where s∈{L|C} indexes the frontend LiDAR and visual observation types, $r_{s}\left(\delta x\right)$ denotes the residual corresponding to observation type s, and $P_{s}$ denotes its associated weighting matrix. The loop-closure constraint acts only at a low frequency on the current keyframe and its active local submap, rather than injecting large historical errors into a single ESIKF state at once. For a geometrically verified loop closure, the SE(3) residual norm is first checked: small residuals can be directly used as low-frequency observation updates; when the residual exceeds the local linearization threshold, the correction is decomposed into K increments, and the same rigid-body correction is simultaneously applied to the active local map to reduce instantaneous inconsistency between the map and the current state. Cross-correlations among frozen historical keyframes are not fully maintained, so this scheme is a real-time-oriented approximate correction and is not equivalent to full pose-graph/smoothing optimization.

The applicability boundary of this approximation is a scenario with moderate loop-closure error where the active submap can be coordinated by rigid-body incremental corrections. In cases of very large-scale loop closures, strong nonrigid map errors, or frequent multiple loop closures, full graph optimization/smoothing provides better consistency. This limitation is listed as future work in Section 7.

## 6. Experimental Design and Results Analysis

### 6.1. Experimental Platform and Validation Procedure

**Figure 7:**
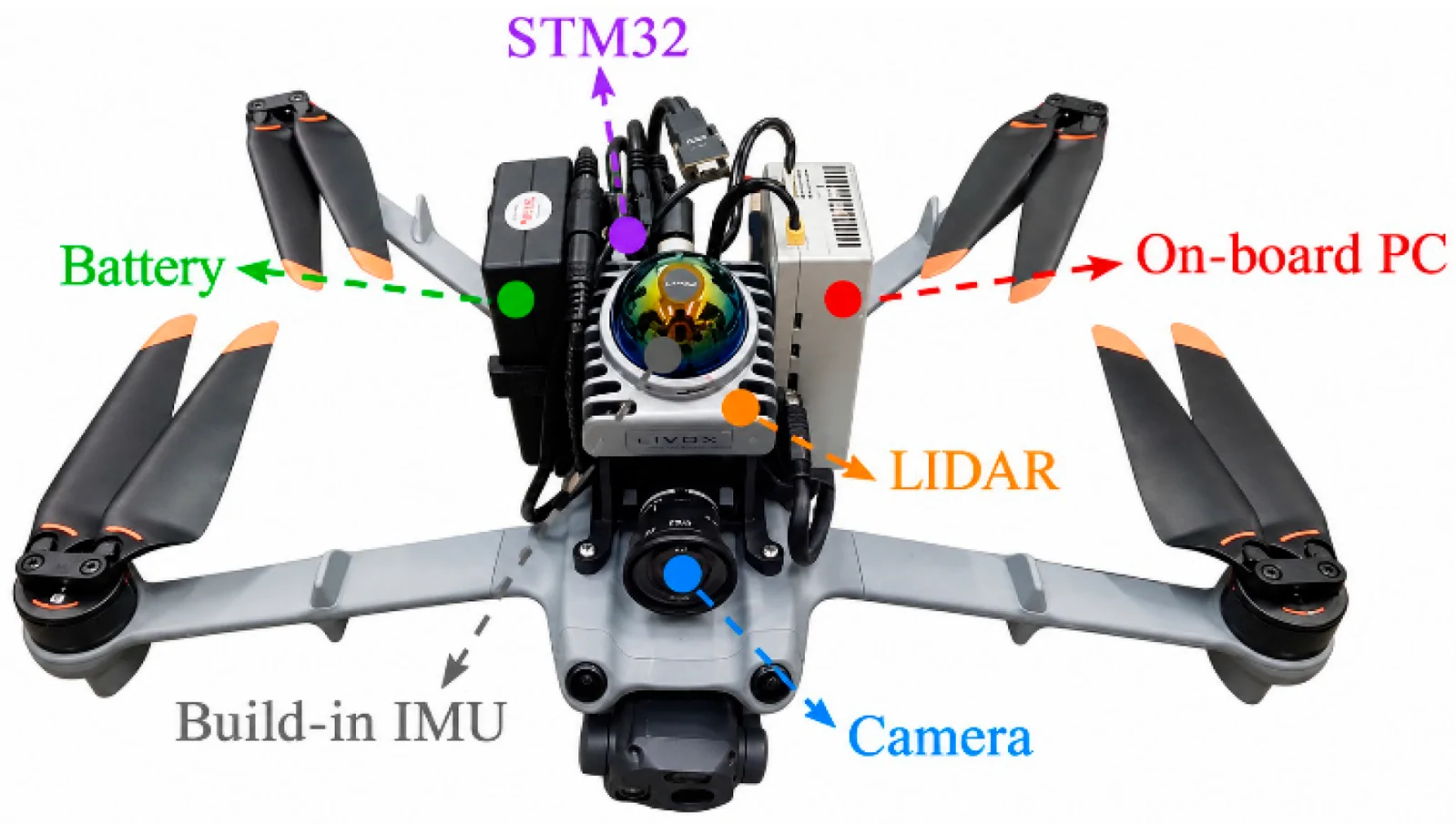
Embedded UAV platform.

The experimental data were collected using a DJI Matrice 3TD UAV (Da-Jiang Innovations Science and Technology Co., Ltd., Shenzhen, China) equipped with sensors and computing hardware. The onboard embedded perception and computing platform is shown in Figure 7.

The models and key parameters of the components are summarized in Table 1.

To ensure comparability across algorithms, all accuracy and ablation experiments were reproduced offline on a desktop PC equipped with an Intel i5-12600KF(Intel, Santa Clara, USA), NVIDIA RTX 4070 Super(NVIDIA, Santa Clara, USA), and 32 GB RAM, running Ubuntu 20.04 and ROS Noetic. Computational-efficiency tests were additionally conducted on an NVIDIA Orin NX to evaluate onboard real-time performance. For comparison methods, the authors’ public implementations and recommended parameters were used whenever possible, with the same ROS data stream, the same ground-truth alignment, and the same evo evaluation pipeline; their core frontends were not rewritten.

Experiments were organized from local to global levels: the joint photometric model, LiDAR degradation quantification and virtual constraints, time-offset correction, and loop-closure retrieval modules were first validated individually; combined and full-system comparisons were then conducted on the self-built datasets and the public NTU and HILTI datasets. Visual and geometric degradation experiments were used to isolate the evaluation of the degradation-robust frontend, while the time-offset and loop-closure experiments were used to evaluate the spatiotemporal-consistency modules. All modules were enabled in the final multi-scenario experiments.

### 6.2. Experiments in Visually Degraded Scenarios

**Figure 8:**
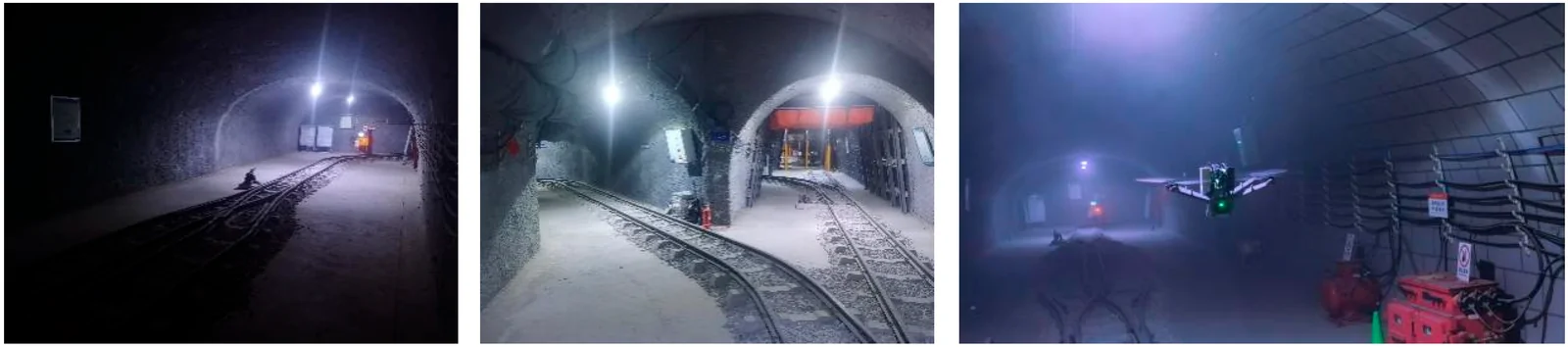
Photograph of the simulated mine scenario.

Two route datasets (A1 and A2) were collected in a simulated mine, while RTK real-time positions and illuminance measured by a lux meter were recorded synchronously. The environment exhibits large illumination variations and weak visual texture, representing a typical visually degraded scenario, as shown in the photograph in Figure 8.

**Figure 9:**
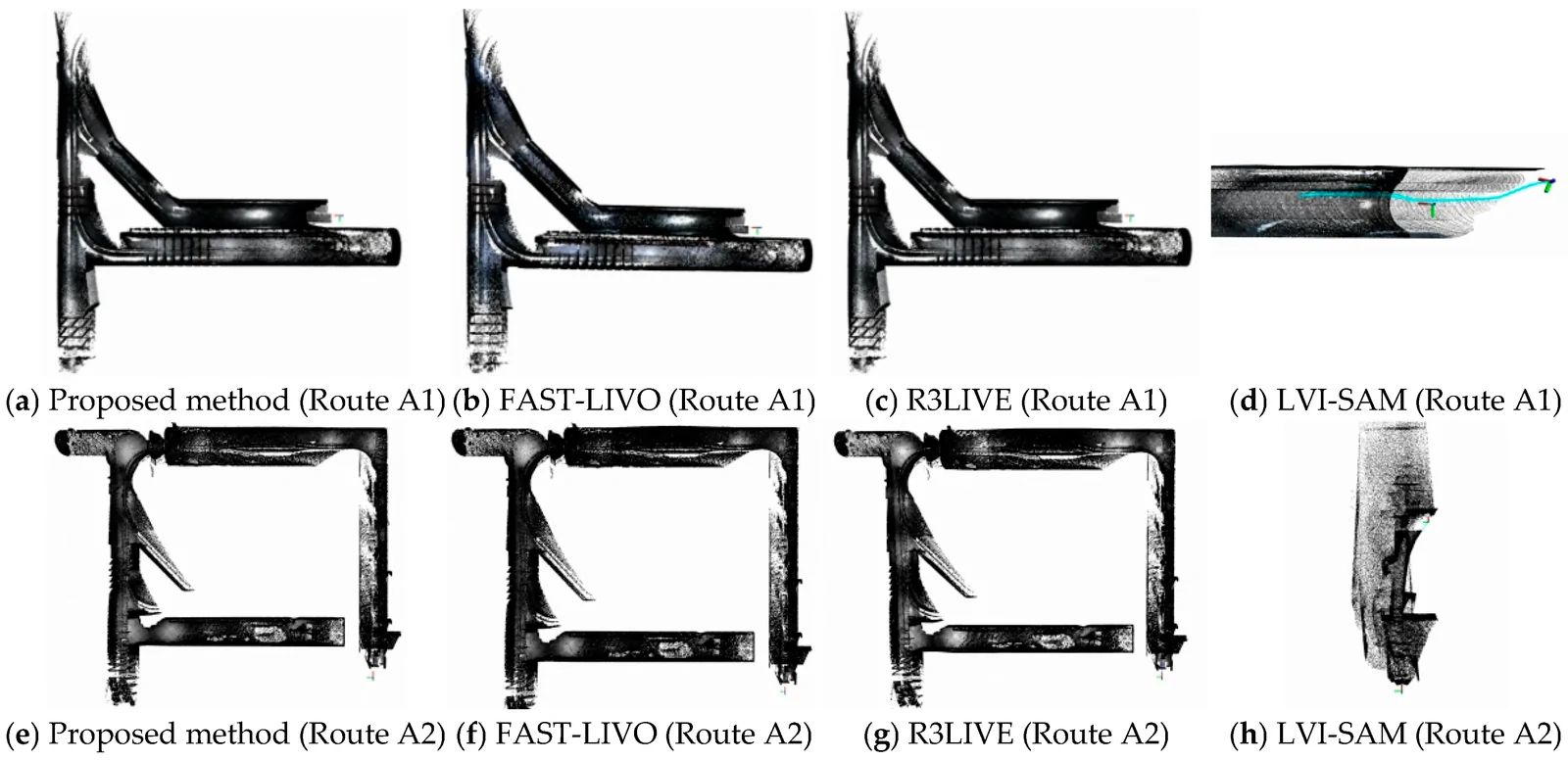
Comparison of mapping results of four algorithms.

Based on Rviz visualization, the mapping performance of the proposed method, FAST-LIVO, R3LIVE, and LVI-SAM on routes A1 and A2 is compared in Figure 9.

**Figure 10:**
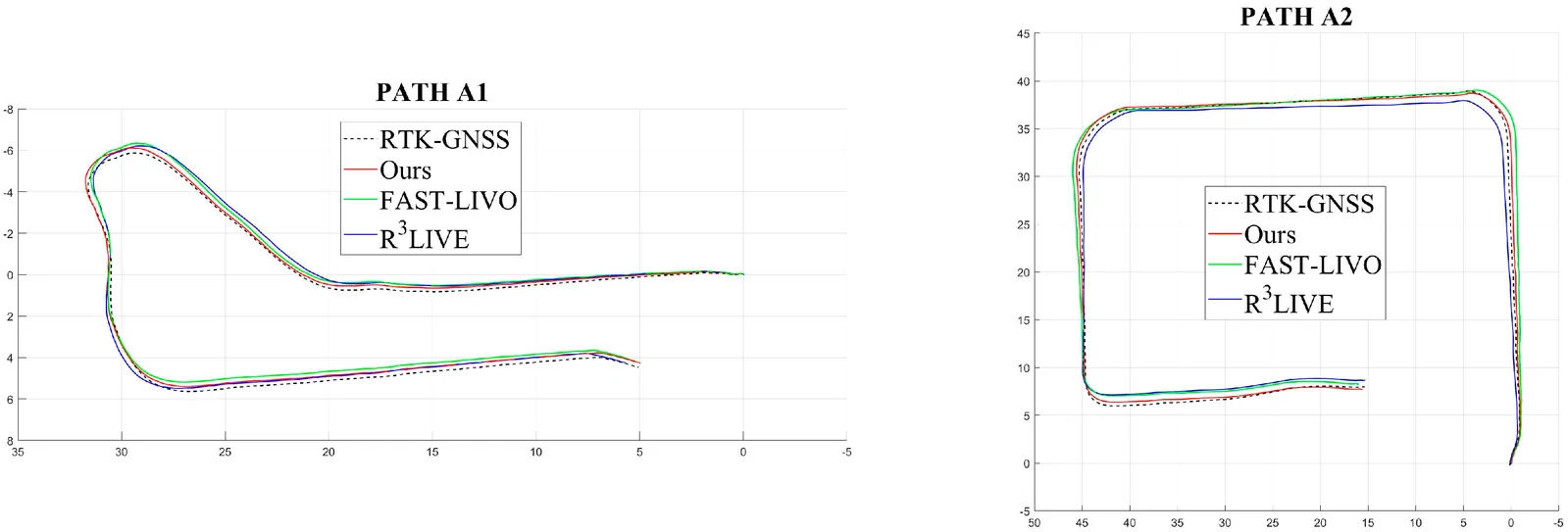
Trajectory comparison of the three algorithms.

The mapping results show that LVI-SAM, which relies on conventional feature-point matching, fails to initialize and diverges in this type of scenario because feature extraction fails. Therefore, subsequent quantitative localization comparisons are performed only for the proposed method, FAST-LIVO, and R3LIVE. The global trajectories of the three algorithms and the RTK-GNSS reference trajectory are compared in Figure 10.

**Figure 11:**
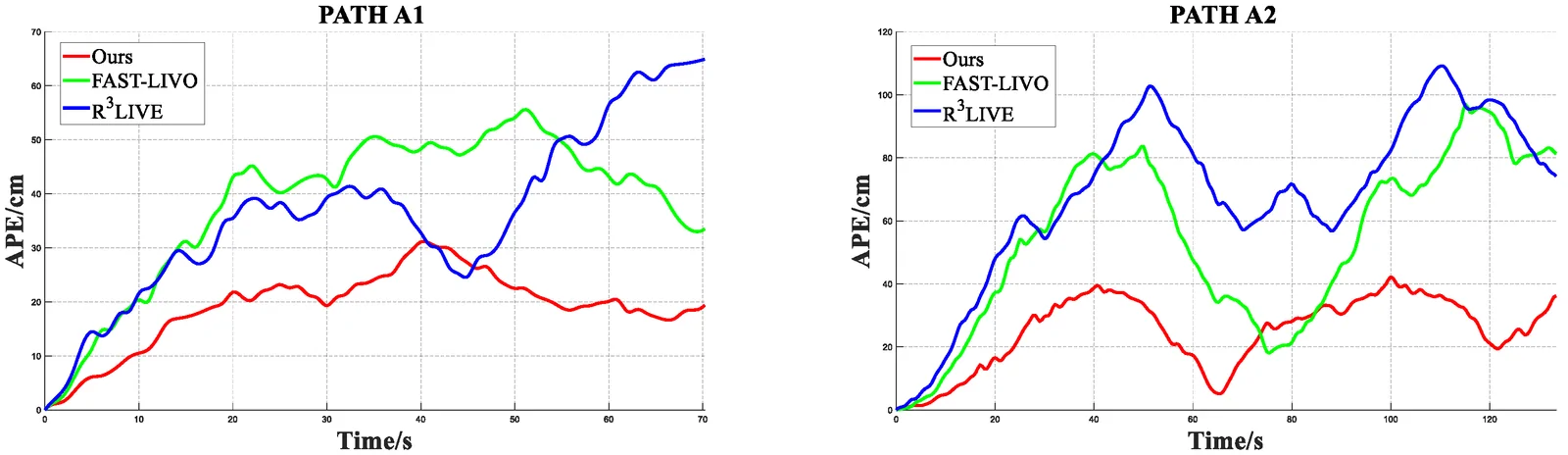
Comparison of absolute pose errors.

Figure 11 presents the real-time absolute pose error (APE) of the three algorithms. In the combined statistics over the two routes, the proposed method achieves an average APE of 0.251 m, compared with 0.543 m for FAST-LIVO and 0.688 m for R3LIVE; its maximum APE is 0.422 m, compared with 0.958 m for FAST-LIVO and 1.091 m for R3LIVE.

**Figure 12:**
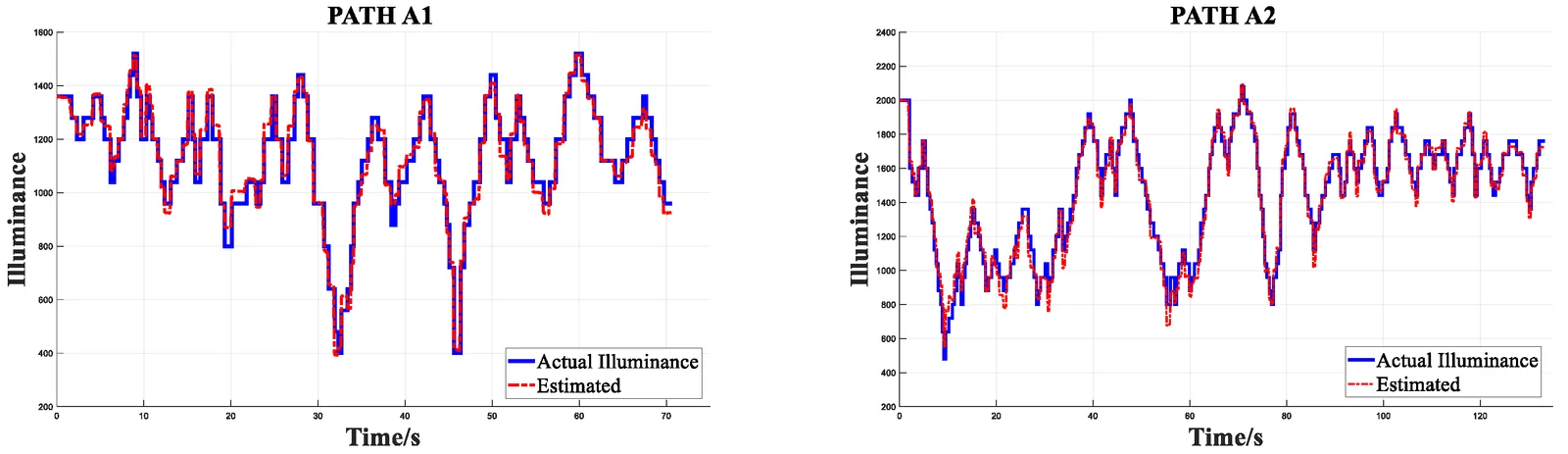
Irradiance prediction results for routes A1 and A2.

In addition, the inverse-mapping performance of the joint photometric model is evaluated in Figure 12. The lux-meter readings, after temporal alignment, are used as an external reference only to determine a single scale factor from relative irradiance to illuminance. Here, “similarity consistency” is explicitly defined as 1-MRE (mean relative error over time). The original experimental MRE is 7.41%, and therefore the corresponding similarity consistency is 92.59%. This metric reflects relative trend consistency after scale alignment and does not represent absolute radiometric calibration accuracy.

### 6.3. Experiments in Lidar-Degraded Scenarios

**Figure 13:**
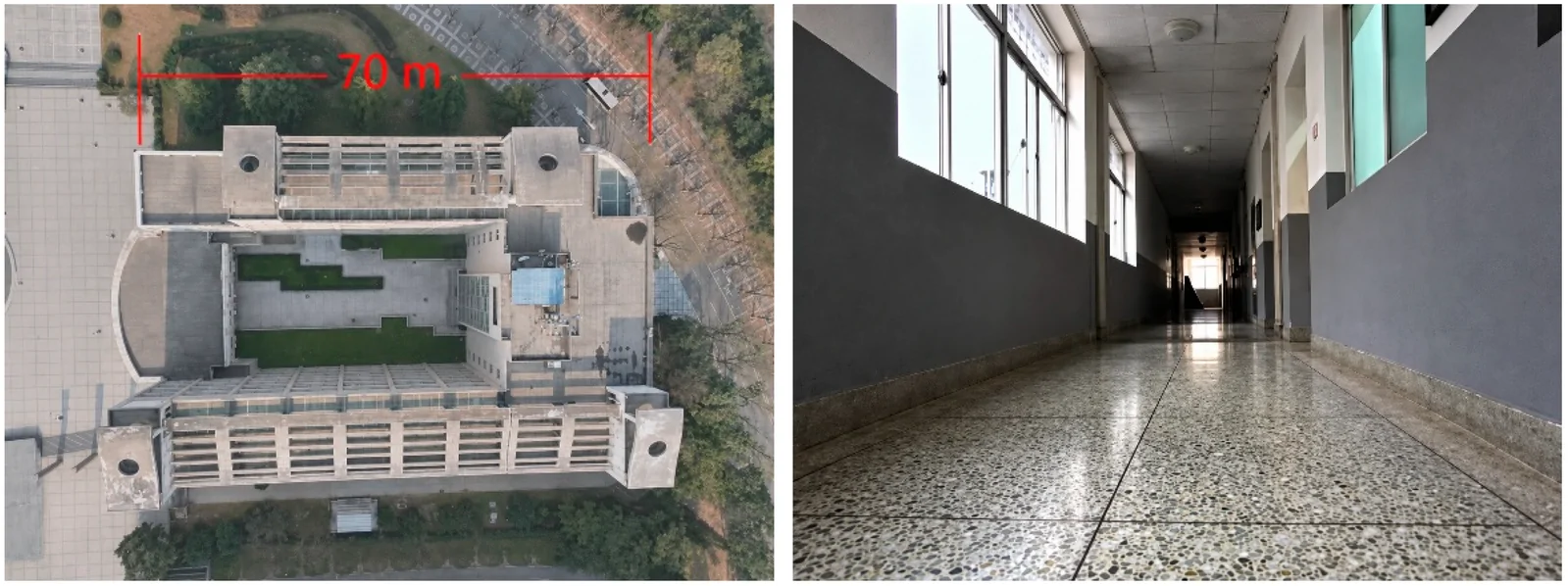
Photograph of the teaching-building scenario.

Dataset B was collected in a teaching building with loop-closure structures. The scene is dominated by large planar walls and glass facades, whose geometric and reflective properties can easily cause LiDAR information degradation, making it a typical LiDAR-degraded environment, as shown in the photograph in Figure 13.

**Figure 14:**
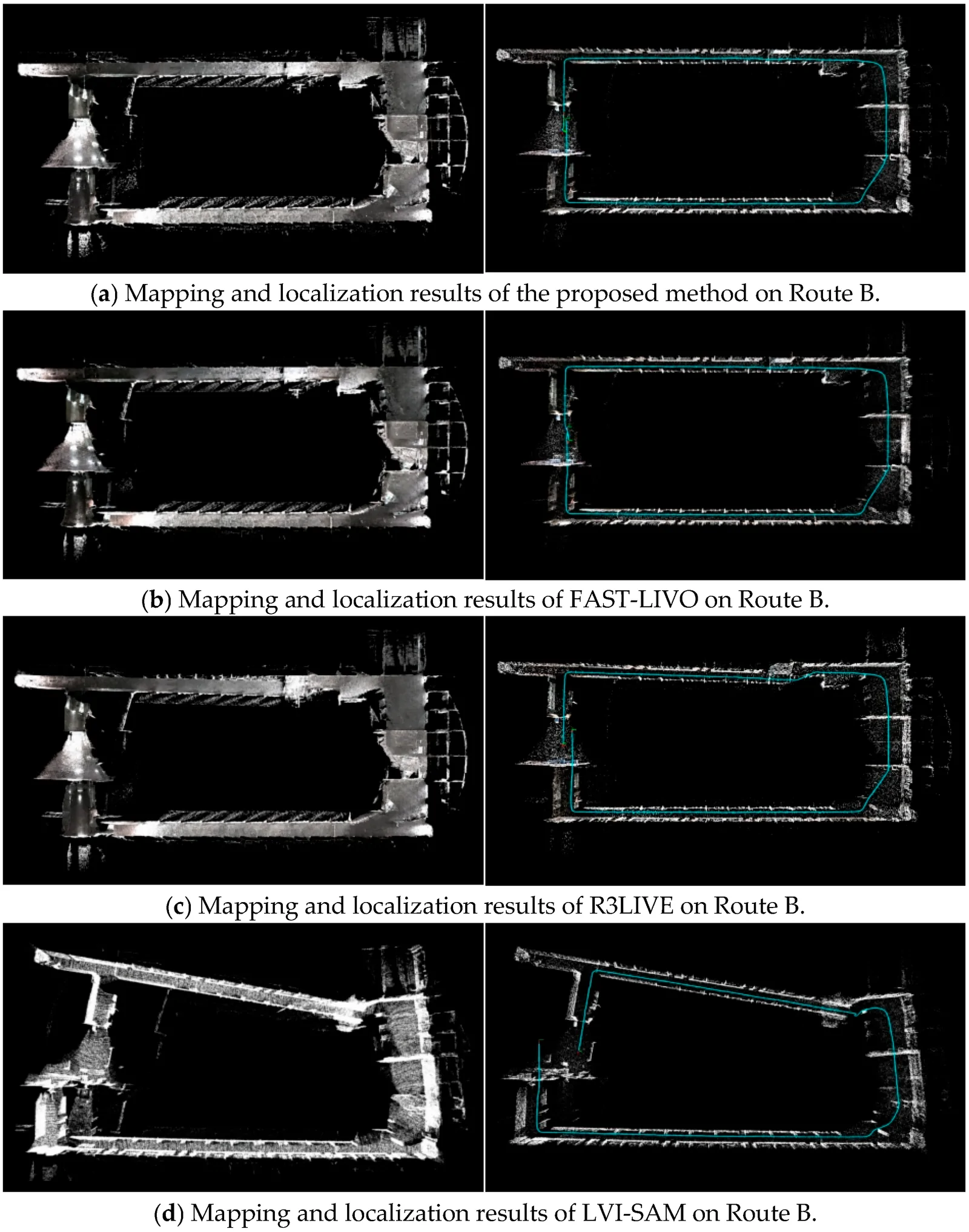
Comparison of mapping and localization results. The blue lines indicate the estimated vehicle trajectories.

Figure 14 shows the global map and global trajectory comparison results obtained in Rviz. The overlap region between the start and end points and the locations with significant trajectory drift are also marked. Because the virtual-constraint fusion strategy is introduced during LiDAR degradation, trajectory drift and oscillation are substantially reduced for the proposed method compared with the comparison methods.

**Figure 15:**
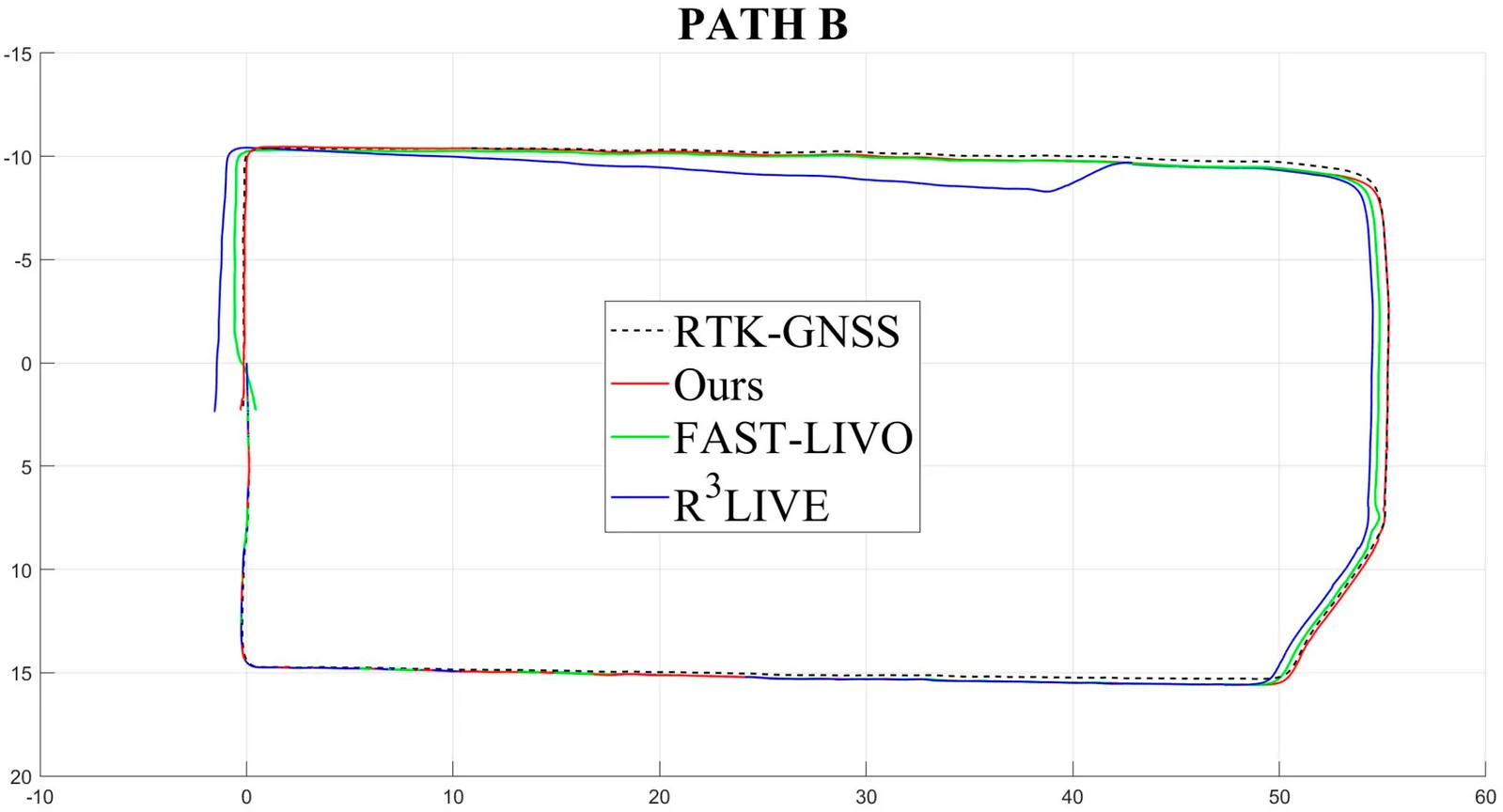
Global trajectory comparison of different algorithms.

The global localization results of the different methods are compared with the RTK-GNSS trajectory in Figure 15. Considering both the start-point deviation and path smoothness, the proposed method shows stronger stability and adaptability in this degraded scenario.

**Figure 16:**
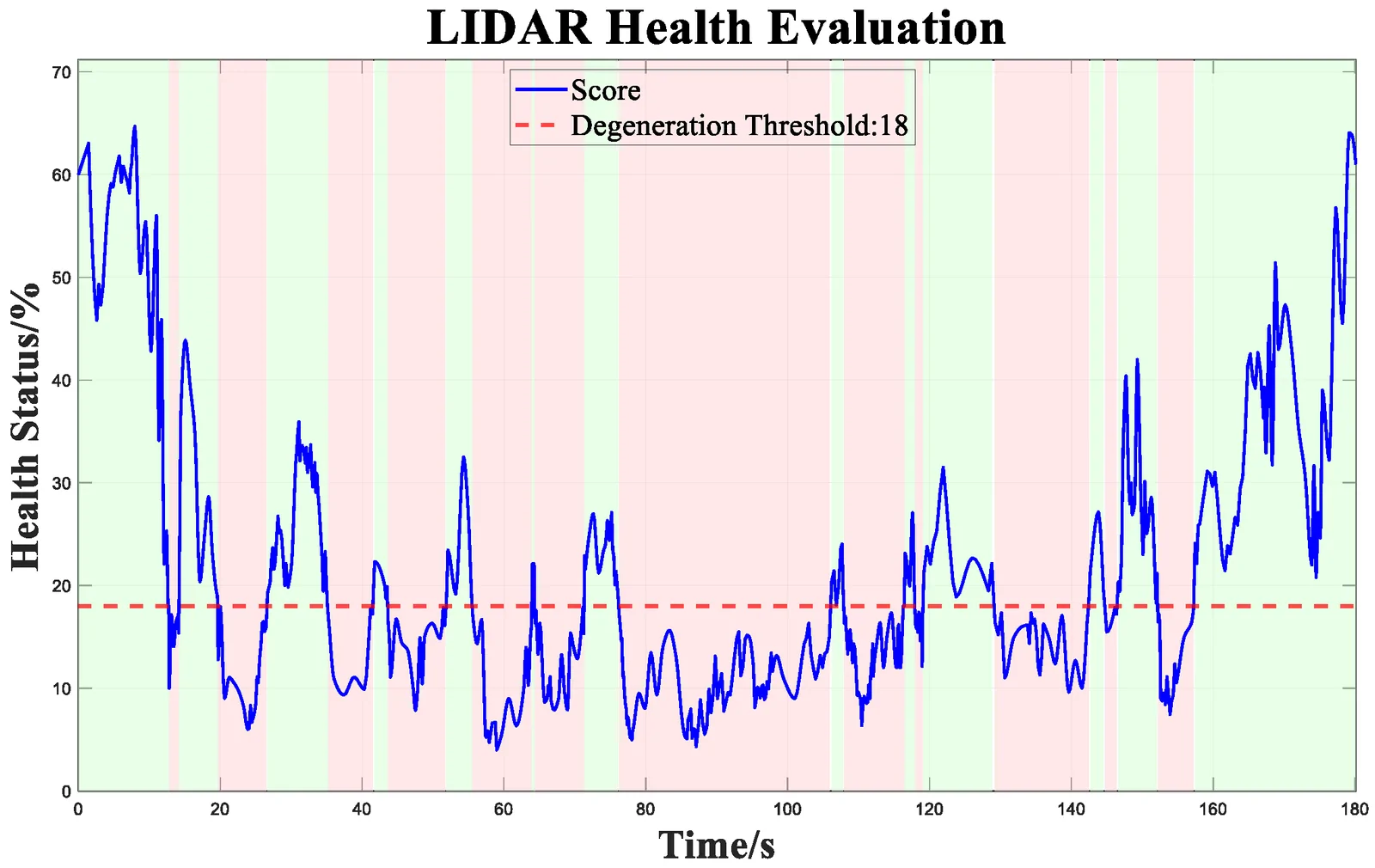
Real-time LiDAR health curve.

Figure 16 presents the scale-normalized real-time LiDAR health. The 18% threshold is not a theoretical constant but a working point obtained from degradation annotations and threshold scanning on the development set: the degradation reference interval is generated jointly from abrupt increases in RTK trajectory error, weak Hessian directions, and manual scene inspection, and a scan is performed over 12–24%. In the experiments, the F1 scores for thresholds of 12%, 15%, 18%, 21%, and 24% are approximately 0.85, 0.91, 0.94, 0.91, and 0.87, respectively, with 18% providing a relatively good balance between missed detections and false triggers. In the main experiment, the cumulative duration below 18% is 97 s. Because this threshold remains related to sensor noise and scene statistics, it should be re-estimated from a small number of calibration sequences when transferred across platforms.

The sensitivity tests further varied point-cloud density (100%, 75%, 50%), measurement-covariance scale, and Sigmoid slope. After whitening and characteristic-scale normalization, the mean health value at the 18% working point varied by about ±3.2 percentage points, while localization RMSE changed by about ±5.6%, indicating that normalization can reduce, but cannot completely eliminate, the influence of sensor and parameter changes.

### 6.4. Validation of the Time-Offset Correction Mechanism

**Figure 17:**
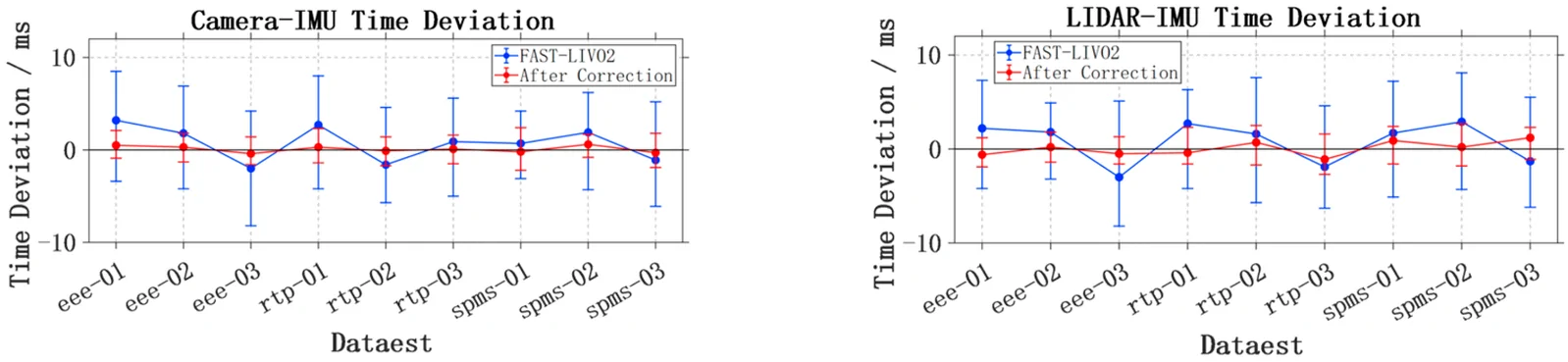
Time-offset correction results.

To verify the effectiveness of the proposed multisource time-offset correction method, the raw sensor data streams and timestamp information were further recorded from ROS on the NTU dataset. During statistics, the IMU timestamp was used as the unified reference time base. For each image and point-cloud frame, the time difference relative to the temporally nearest IMU measurement was extracted, and the maximum, minimum, and range of the Camera–IMU and LiDAR–IMU time offsets were calculated over the complete sequences of each dataset. This module focuses on the correction capability of time-offset estimation and timestamp remapping for multisource synchronization errors. The comparison of time-offset ranges before and after time-offset correction is shown in Figure 17. The blue curves represent the original FAST-LIVO2 results, while the red curves represent the corrected results after introducing online time-offset estimation and timestamp remapping.

The experimental results show that the ranges of the corrected Camera–IMU and LiDAR–IMU time offsets converge significantly overall; the red curves are closer to zero offset across all datasets, and their fluctuation amplitudes are smaller than those of the original results. These results indicate that estimating the LiDAR- and camera-to-IMU time offsets online as filter states can effectively suppress multisource temporal asynchrony and improve consistency between the observation construction time and the IMU propagation state.

### 6.5. Independent Validation of the Keyframe Strategy and Loop-Closure Module

#### 6.5.1. Keyframe Parameter Selection

**Figure 18:**
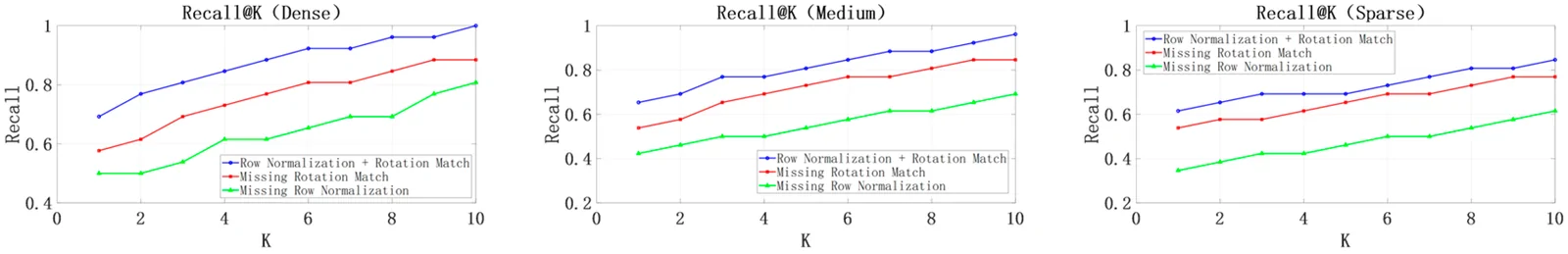
Recall comparison under three keyframe strategies.

**Figure 19:**
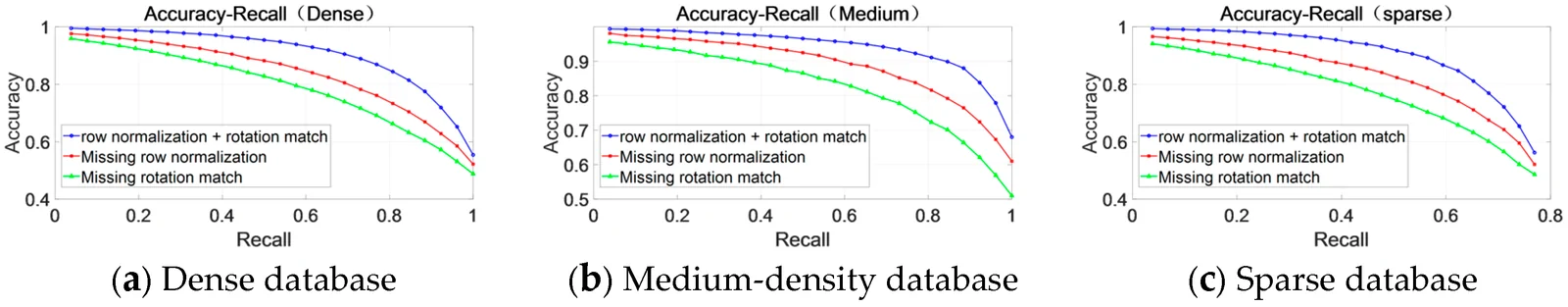
Precision–recall comparison under three keyframe strategies.

First, to determine the keyframe-selection parameters of the loop-closure detection module, 26 representative loop-closure points were selected from the NTU dataset for experimental validation, and three keyframe databases with dense, medium, and sparse densities were constructed. By comparing Recall@K curves and precision–recall curves under the different database conditions, the influence of keyframe-density variation on loop-closure candidate coverage, matching accuracy, and performance stability was evaluated. The experimental results are shown in Figure 18 and Figure 19.

Overall, Figure 18 and Figure 19 show that different keyframe densities simultaneously affect the stability of the Recall@K and precision–recall curves. Although the dense database provides more candidate frames, it also introduces interference from similar frames, causing precision to decrease more noticeably in the high-recall region; the sparse database reduces the candidate-set size but lacks sufficient valid loop-closure candidates, limiting the upper bound of recall. In comparison, the medium-density database provides a relatively good balance between the number of candidates and matching quality, allowing both types of curves to maintain relatively stable performance. Therefore, the medium threshold (2.0 m/20°) is selected as the main experimental parameter in subsequent experiments.

#### 6.5.2. Independent Validation of the Loop-Closure Module

After determining the keyframe thresholds, to independently validate the ability of the loop-closure detection module to suppress cumulative drift, loop-closure detection and constraint optimization were added to FAST-LIVO2 without introducing the time-offset correction mechanism. A building scenario with a typical revisit path was selected, with particular attention to trajectory start-end closure consistency and changes in ATE.

**Figure 20:**
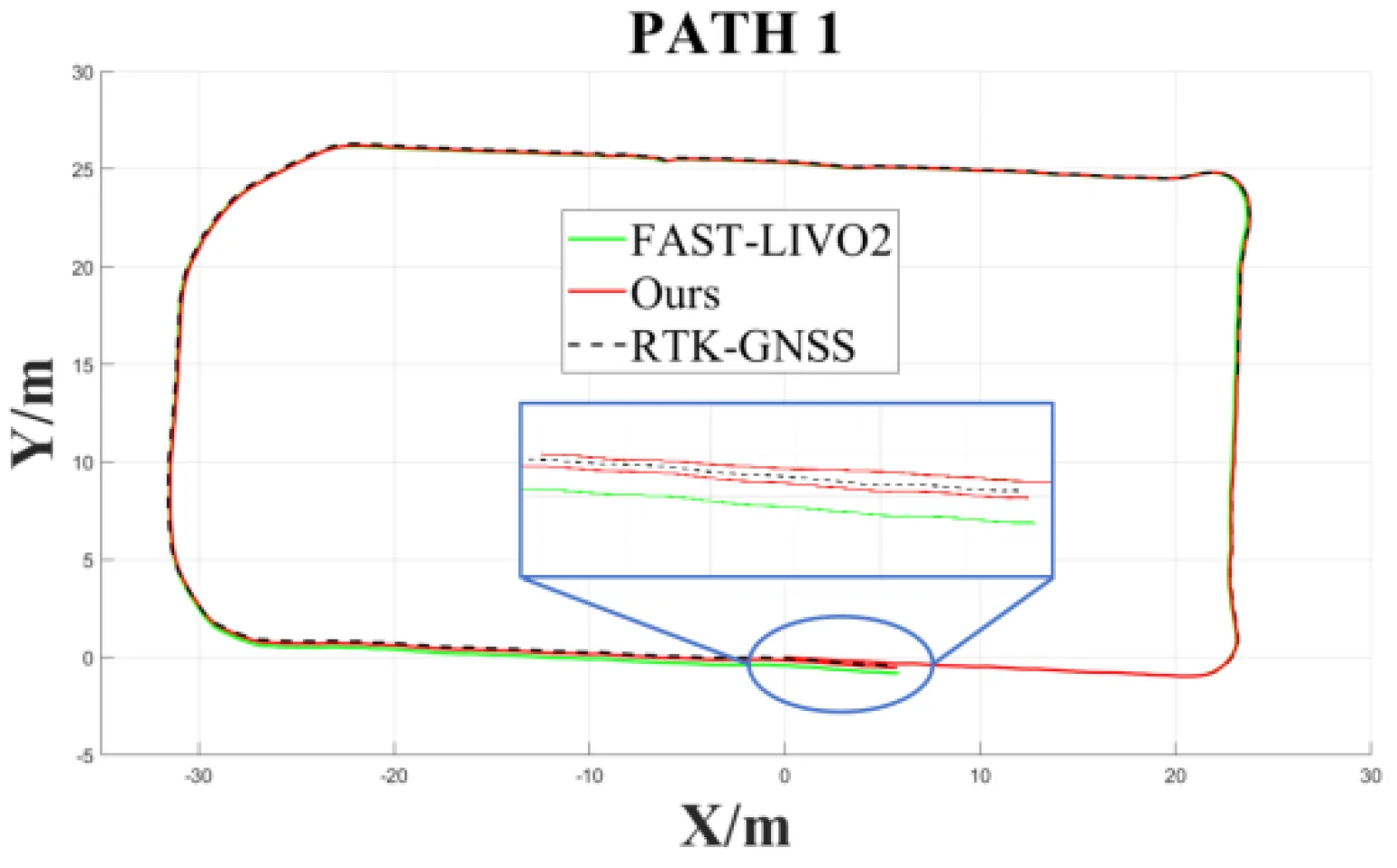
Loop-closure trajectory comparison. the blue inset shows a zoomed-in view of the circled region to highlight the trajectory differences among the compared methods.

After processing the offline data and visualizing the trajectories in MATLAB (R2025b), the comparison results shown in Figure 20 were obtained. Compared with FAST-LIVO2, the addition of the loop-closure detection module yields higher start-end closure consistency and significantly reduces the start-to-end deviation.

**Figure 21:**
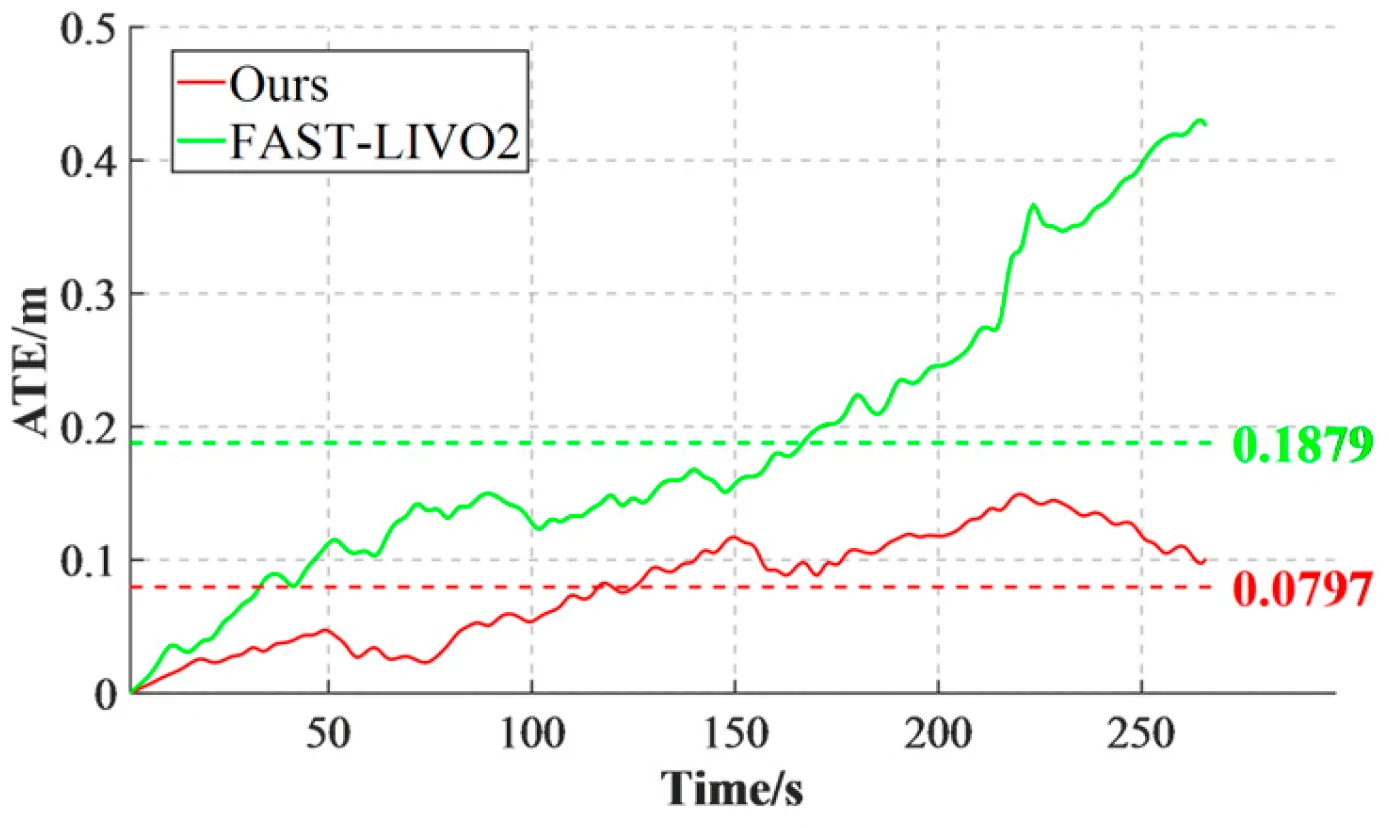
ATE comparison of two algorithms.

Figure 21 compares the absolute trajectory error (ATE) of the two methods. The proposed method achieves an average ATE of 0.0797 m, a reduction of 0.1082 m compared with 0.1879 m for FAST-LIVO2. Meanwhile, in the later part of the trajectory, the localization error of the proposed method changes from continuous accumulation to gradual convergence, indicating that the loop-closure constraint can use historical revisit information to correct cumulative drift during long-term operation.

### 6.6. Comprehensive Validation of Combined Modules and the Full System

#### 6.6.1. Comprehensive Comparison on the Self-Built Degradation Dataset

To further quantify localization performance, trajectory root-mean-square error (RMSE) was analyzed for the above three self-built dataset experiments, and the statistical results are listed in Table 2.

As shown in Table 2, under the same self-built dataset conditions, the proposed method exhibits lower RMSE than the direct baseline FAST-LIVO2 as well as FAST-LIVO, R3LIVE, and LVI-SAM. The inclusion of FAST-LIVO2 allows the improvement attributable to the added modules to be more directly separated from differences in the parent framework.

#### 6.6.2. Combined Validation on Typical Loop-Closure Sequences from Ntu

**Figure 22:**
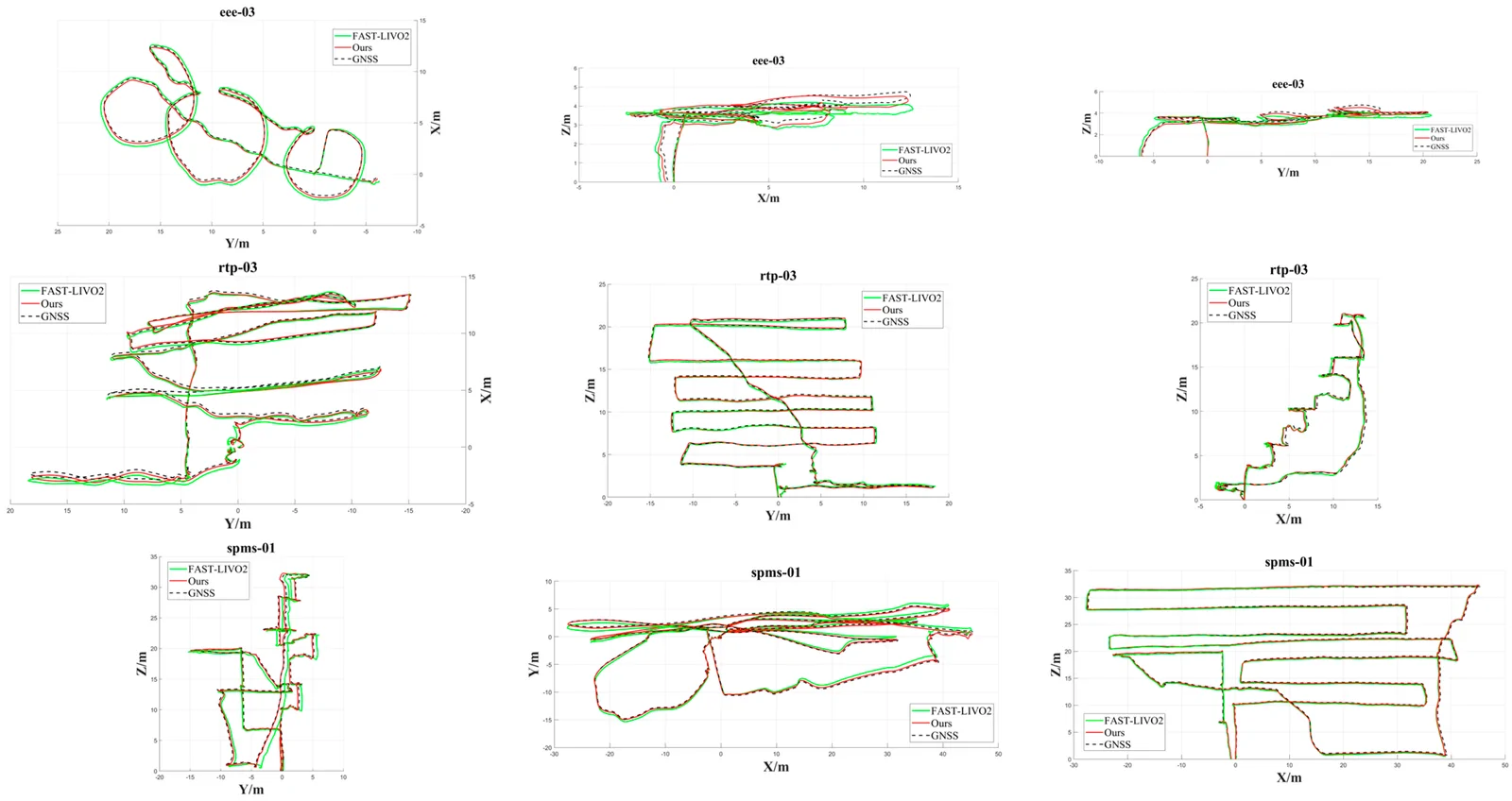
Trajectory comparison of different algorithms on the respective sequences.

After independently validating the loop-closure module and the time-offset correction mechanism, both were introduced into the system, and multiple sequences with typical loop-closure trajectories were selected from the NTU dataset for comprehensive trajectory-error comparison. The three-view global trajectory comparisons are shown in Figure 22. From top to bottom, the three subfigures correspond to the X-Y, X-Z, and Y-Z plane views, respectively. The black dashed line represents the ground-truth trajectory, the red trajectory is the estimate from the proposed method, and the green trajectory is the estimate from FAST-LIVO2.

**Figure 23:**
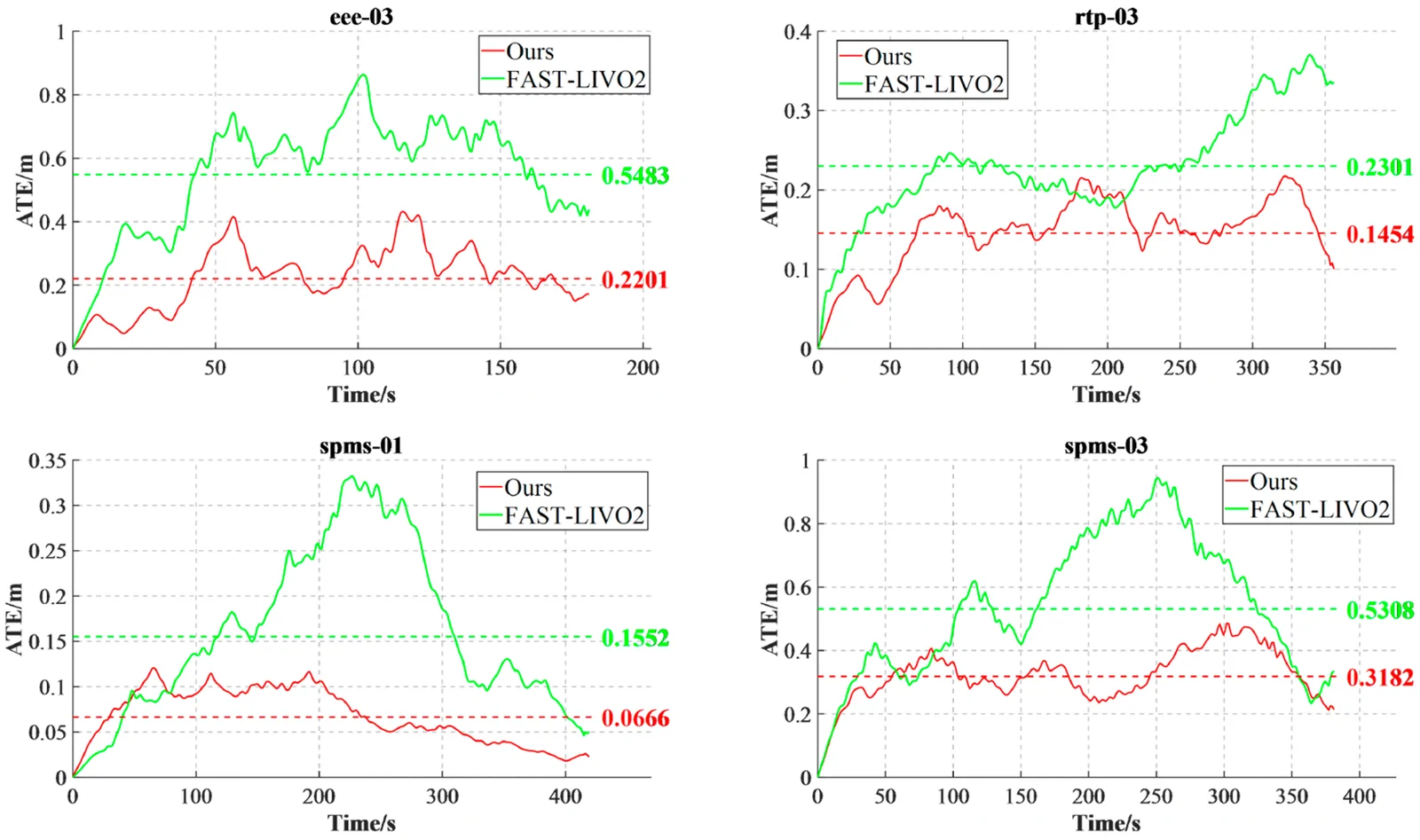
ATE comparison on four sequences.

Figure 23 shows the ATE comparison of the two methods on four sequences. Compared with FAST-LIVO2, the proposed method obtains lower localization error on the eee_03, rtp_03, spms_01, and spms_03 sequences, with an average ATE reduction of 0.188 m, indicating that time-offset correction and loop-closure constraints jointly suppress error accumulation in long sequences with revisit structures. This result further indicates that time-offset correction improves multisource observation-alignment consistency, while loop-closure constraints further suppress long-term cumulative drift; together, the two mechanisms improve overall trajectory accuracy and stability.

#### 6.6.3. Multi-Scenario Generalization Validation on Hilti and Ntu

To distinguish the gains from the degradation-robust frontend from those of the complete spatiotemporal-consistency system, the frontend version with only photometric consistency and degradation virtual constraints enabled is first evaluated on nine HILTI sequences, followed by enabling time-offset correction and loop closure on additional NTU and HILTI sequences. This progressive design avoids conflating local robustness improvements with global loop-closure correction.

**Figure 24:**
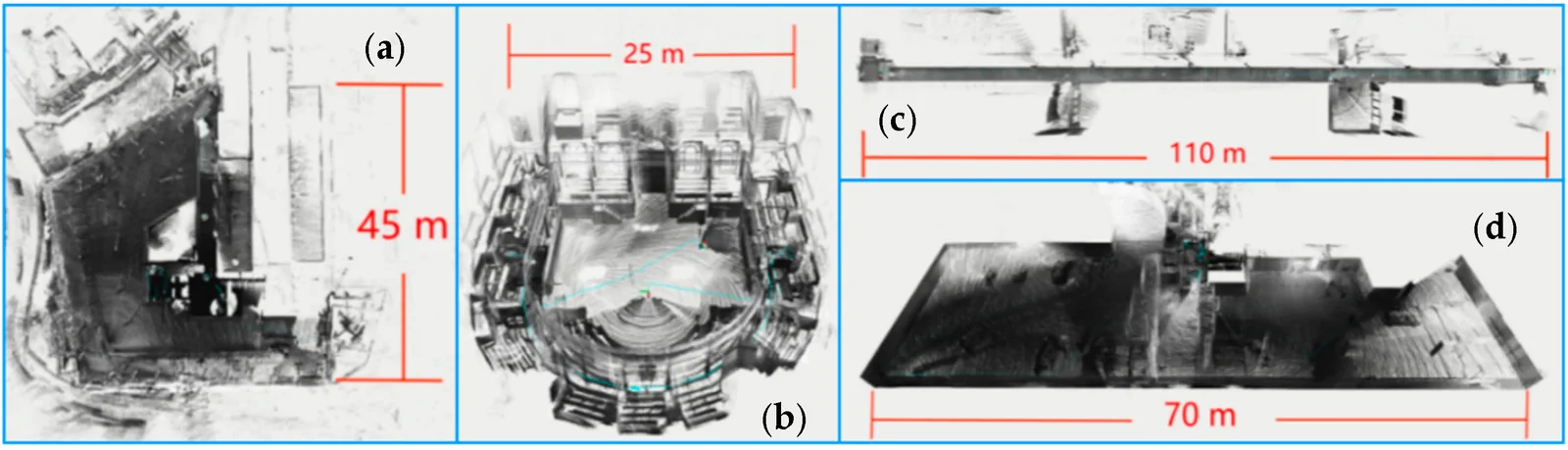
Mapping results on the HILTI dataset. (**a**) Construction site; (**b**) auditorium; (**c**) corridor; (**d**) building staircase.

Given the limited coverage of the self-built datasets, the method is further evaluated on nine sequences from the public HILTI SLAM Challenge dataset, covering construction sites, auditoria, corridors, and underground parking lots. The mapping results are shown in Figure 24.

The localization RMSE results on the public dataset are presented in Table 3.

#### 6.6.4. Module Ablation Under Unified Conditions

To quantify the independent contribution of each module, controlled ablation experiments were performed under the same validation set, the same FAST-LIVO2 code baseline, and the same evaluation pipeline, using both “single-module addition” and “full-system” configurations. The results are shown in Table 4.

The ablation results show that all four modules provide gains, and the full system reduces the RMSE from 0.153 m for FAST-LIVO2 to 0.117 m, corresponding to an approximately 23.5% reduction. Loop closure and degradation constraints contribute more noticeably in long-term drift/weak-observability scenarios, while time correction and the photometric model mainly improve observation consistency. The full system performs better than any single module, supporting the conclusion that the modules are complementary rather than fully redundant.

#### 6.6.5. Computational Efficiency and Onboard Real-Time Performance

To quantify the computational cost of the added modules, runtime statistics were collected on both a desktop platform and the Orin NX. The results are shown in Table 5. The loop-closure module runs in a low-frequency thread, and its time is amortized per frame.

At the 10 Hz LiDAR main-update rate, the results show that the Orin NX full system still retains an equivalent processing capability of about 16.2 Hz, leaving real-time headroom. Compared with FAST-LIVO2, the added time-offset estimation, photometric processing, degradation assessment/virtual observations, and low-frequency loop closure increase the average per-frame computational cost by about 12.1 ms. This result supports real-time operation only under the current sensor frequencies and implementation version; it is not extrapolated to higher-resolution cameras or higher-frequency point clouds.

## 7. Conclusions and Limitations

This paper addresses the stability of tightly coupled LiDAR–Camera–IMU localization and mapping systems for UAVs in complex degraded environments and proposes an enhanced framework that jointly strengthens observation consistency at the frontend and global constraints at the backend.

For multisource temporal consistency, the time offsets of LiDAR and the camera relative to the IMU are incorporated into the filtering framework as state variables, with their slowly varying characteristics modeled by random walks, and an estimable relationship is established through the Jacobian of the observation residual with respect to the time offsets. After filter updates, the estimated time offsets are fed back to the point-cloud sampling time and image exposure time, realizing a closed-loop correction of “online estimation–timestamp remapping–observation reconstruction–filter update.” Experimental results show that the ranges of the Camera–IMU and LiDAR–IMU time offsets converge overall after correction and that the fluctuation amplitudes are significantly reduced, indicating that the mechanism effectively improves temporal consistency between multisource observations and the IMU propagation state.

In visually degraded scenarios, to address the influence of lens vignetting, exposure changes, and nonlinear camera response on direct visual residuals, a joint photometric model is constructed. Scale gauge freedom is removed through reference exposure, central vignetting, and CRF endpoint constraints, while feasible-domain projection guarantees positive vignetting and monotonicity of the inverse CRF. In the simulated mine experiment, the proposed method achieves an average APE of 0.251 m; the average relative error under the external illuminance reference is 7.41%, corresponding to a defined similarity consistency of 92.59%.

In LiDAR geometric degradation scenarios, the six-degree-of-freedom Jacobian is first whitened and rotation-translation scale-normalized, after which a health indicator is constructed from the Hessian spectrum. The 18% threshold comes from threshold scanning on the development set rather than being a fixed theoretical constant. Virtual observations are jointly gated by direction selection, steady-state confidence, and innovation thresholds to avoid incorrectly constraining legitimate lateral/vertical multirotor maneuvers.

For global consistency, a Scan Context-type descriptor is used to generate low-frequency loop-closure candidates, and cumulative drift is suppressed through geometric verification, adaptive covariance, small-residual gating, and progressive correction. This strategy preserves the implementation simplicity of the filtering frontend, but does not explicitly maintain all cross-correlations among historical states as a full pose-graph/smoothing method would.

The comprehensive experiments and unified ablation show that the individual modules respectively improve temporal alignment, photometric consistency, constraints along degenerate directions, and long-term drift, and exhibit complementary behavior in the full system.

Limitations and scope of applicability: ① Time offsets may remain locally unobservable under low motion excitation combined with weak texture and geometric degradation; the current approach freezes updates rather than fundamentally eliminating unobservability. ② The photometric model recovers only relative irradiance; severe saturation, automatic white balance, and spatially nonuniform exposure may still violate the model. ③ The 18% health threshold is not universally transferable across sensors and requires a small amount of calibration data. ④ Direction-selective kinematic constraints are not applicable to sustained large lateral/vertical accelerations or strong-wind maneuvers. ⑤ Filter-based loop-closure correction ignores full historical cross-correlations and is less globally consistent than full smoothing under very large loop closures. ⑥ The ring-sector maximum-height descriptor may still be affected by dynamic occlusion and structural changes. ⑦ The current onboard real-time conclusion is limited by sensor frequency, resolution, and the degree of implementation optimization. Future work will focus on adaptive thresholds, semantic removal of dynamic-scene interference, and a hybrid filter-graph-optimization backend.

## Figures and Tables

**Table 1:** Hardware parameters.

Sensor	Model	Basic Parameters
Onboard computer	NVIDIA Orin NX(NVIDIA, Santa Clara, USA)	6-core CPU, 1024 CUDA cores
LiDAR	Livox MID-360(Livox Technology Co., Ltd., Shenzhen, China)	200,000 points/s
Camera	Hik MV-CA013-A0UC(Hikrobot (Hikvision Robotics), Hangzhou, China)	Resolution: 1280 × 1024; frame rate: 10–60 Hz
IMU	ICM40609(InvenSense (TDK), San Jose, USA)	6 degrees of freedom
Synchronizer	STM32(STMicroelectronics, Geneva, Switzerland)	—

**Table 2:** Trajectory RMSE comparison of five algorithms on the self-built datasets (unit: m; “×” indicates that the algorithm failed to complete valid initialization or diverged).

Sequence	Proposed Method	FAST-LIVO	R3LIVE	LVI-SAM	FAST-LIVO2
PATH A1	0.244	0.420	0.449	×	0.302
PATH A2	0.415	0.691	0.856	×	0.501
PATH B	0.480	0.858	0.743	4.362	0.616
Average	0.379	0.656	0.683	4.362	0.473

**Table 3:** Trajectory RMSE comparison of multiple algorithms on HILTI sequences (unit: m; “×” indicates failure to complete valid initialization, runtime divergence, or failure to obtain a trajectory suitable for evaluation).

Sequence	Ours	FAST-LIVO	R^3^LIVE	LVI-SAM	FAST-LIO2	FAST-LIVO2
site1_handheld_1	0.021	0.025	0.021	×	0.033	0.024
site1_handheld_2	0.025	0.024	0.026	8.664	0.024	0.028
site1_handheld_3	0.036	0.045	0.051	×	0.047	0.041
site1_handheld_4	0.015	0.036	0.018	×	0.021	0.018
site1_handheld_5	0.097	0.149	0.121	3.489	0.114	0.122
exp03_construction_stairs	0.194	0.243	0.779	8.878	0.322	0.251
exp07_long_corridor	0.055	0.068	0.063	6.118	0.112	0.081
exp11_lower_gallery	0.017	0.022	0.010	2.283	0.026	0.020
exp16_upper_gallery	0.412	0.623	2.408	×	0.721	0.496
Average	0.097	0.137	0.389	5.886	0.158	0.120

**Table 4:** Performance comparison between individual modules and the full system.

Configuration	Time Correction	Photometric Model	Degradation Constraint	Loop Closure	Average RMSE/m
FAST-LIVO2	—	—	—	—	0.153
Time Offset Only	√	—	—	—	0.145
Photometric Model Only	—	√	—	—	0.139
Degradation Constraint Only	—	—	√	—	0.128
Loop Closure Only	—	—	—	√	0.121
Full System	√	√	√	√	0.117

Note: “√” indicates that the corresponding module is included in the configuration, whereas “—” indicates that the corresponding module is not included.

**Table 5:** Runtime, equivalent processing frequency, and peak memory usage of FAST-LIVO2 and the full system on the desktop and Orin NX platforms.

Platform/Configuration	Average Frontend Time/ms	Amortized Loop-Closure Time/ms	Total Time/ms	Equivalent Frequency/Hz	Peak Memory/GB
Desktop FAST-LIVO2	25.8	—	25.8	38.8	2.2
Desktop Full System	32.1	2.5	34.6	28.9	2.6
Orin NX FAST-LIVO2	49.7	—	49.7	20.1	2.1
Orin NX Full System	57.4	4.4	61.8	16.2	2.5

Note: “—” indicates not applicable because the baseline FAST-LIVO2 does not include the loop-closure module.

## Data Availability

The public data used in this study are available from the HILTI SLAM Challenge dataset. The campus and mine datasets used in this study were collected by the authors and are not publicly available.
